# Differential Analysis of the Nasal Microbiome of Pig Carriers or Non-Carriers of *Staphylococcus aureus*

**DOI:** 10.1371/journal.pone.0160331

**Published:** 2016-08-10

**Authors:** Carmen Espinosa-Gongora, Niels Larsen, Kristian Schønning, Merete Fredholm, Luca Guardabassi

**Affiliations:** 1 Department of Veterinary Disease Biology, Faculty of Health and Medical Sciences, University of Copenhagen, Frederiksberg C, Denmark; 2 Danish Genome Institute, Aarhus, Denmark; 3 Department of Clinical Microbiology, Hvidovre University Hospital, Hvidovre, Denmark; 4 Department of Clinical Medicine, Faculty of Health and Medical Sciences, University of Copenhagen, Copenhagen, Denmark; 5 Department of Veterinary Clinical and Animal Sciences, Faculty of Health and Medical Sciences, University of Copenhagen, Frederiksberg C, Denmark; 6 Department of Biomedical Sciences, Ross University School of Veterinary Medicine, Basseterre, Saint Kitts Nevis, West Indies; Rockefeller University, UNITED STATES

## Abstract

*Staphylococcus aureus* is presently regarded as an emerging zoonotic agent due to the spread of specific methicillin-resistant *S*. *aureus* (MRSA) clones in pig farms. Studying the microbiota can be useful for the identification of bacteria that antagonize such opportunistic veterinary and zoonotic pathogen in animal carriers. The aim of this study was to determine whether the nasal microbiome of pig *S*. *aureus* carriers differs from that of non-carriers. The V3-V5 region of the 16S rRNA gene was sequenced from nasal swabs of 44 *S*. *aureus* carriers and 56 non-carriers using the 454 GS FLX titanium system. Carriers and non-carriers were selected on the basis of quantitative longitudinal data on *S*. *aureus* carriage in 600 pigs sampled at 20 Danish herds included in two previous studies in Denmark. Raw sequences were analysed with the BION meta package and the resulting abundance matrix was analysed using the DESeq2 package in R to identify operational taxonomic units (OTUs) with differential abundance between *S*. *aureus* carriers and non-carriers. Twenty OTUs were significantly associated to non-carriers, including species with known probiotic potential and antimicrobial effect such as lactic acid-producing isolates described among *Leuconostoc* spp. and some members of the *Lachnospiraceae* family, which is known for butyrate production. Further 5 OTUs were significantly associated to carriage, including known pathogenic bacteria such as *Pasteurella multocida* and *Klebsiella* spp. Our results show that the nasal microbiome of pigs that are not colonized with *S*. *aureus* harbours several species/taxa that are significantly less abundant in pig carriers, suggesting that the nasal microbiota may play a role in the individual predisposition to *S*. *aureus* nasal carriage in pigs. Further research is warranted to isolate these bacteria and assess their possible antagonistic effect on *S*. *aureus* for the pursuit of new strategies to control MRSA in pig farming.

## Introduction

The composition of the microbiota is known to directly influence the host’s health and disease. For example the gut microbiota has been shown to play a role in conditions such as intestinal inflammatory bowel disease, obesity or diabetes in humans [1–3]. Similar studies in animals have shown that specific microbiome profiles of the milk predispose to mastitis in dairy cows [4] and specific enterotypes may improve pig productivity traits such as body weight and average daily gain [5]. The study of the microbiota in healthy and diseased livestock can lead to identification of bacteria that antagonize specific animal or zoonotic pathogens. These bacteria may be useful in the prevention and eventually treatment of diseases. In times where the spread of antimicrobial-resistant bacteria in livestock calls for prudent antimicrobial use, probiotics may be used to improve livestock health as an alternative to conventional antimicrobials [6, 7]. Some probiotics have been shown to be promising in the reduction of zoonotic bacteria in livestock such as *Campylobacter jejuni* in broiler chickens [8]. However, this resource remains largely unexplored for control of zoonotic bacteria.

In recent years, there has been an increasing concern about the spread of methicillin-resistant *Staphylococcus aureus* (MRSA) in pig farming [9]. Specific pig-adapted clones such as sequence type (ST)398 are responsible for a considerable fraction of MRSA infections among farm workers, especially in pig-exporting countries with low MRSA prevalence in the human population, such as Denmark [10]. The few intervention studies that have been published on MRSA control in pig farms were based on reduction of antimicrobial use [11] or implementation of hygiene and disinfection programmes [11–13]. However, none of these studies led to conclusive results with the exception of Norway, a country with low prevalence of MRSA in pig farming that implemented a program requiring depopulation and strict disinfection of farms [14]. A recent longitudinal study showed that certain pigs had individual predisposition to *S*. *aureus* nasal colonization [15]. Subsequently this predisposition to *S*. *aureus* carriage in pigs was associated to a specific single nucleotide polymorphism (MARC0099960), possibly associated to functional variants of chemokines [16]. In this study we explored the nasal microbiome of pigs classified as *S*. *aureus* carriers and non-carriers based on the results of a previous longitudinal quantitative study [15]. The aim was to determine whether the nasal microbiome of pig carriers differs from that of non-carriers by studying differential abundance of taxa between the two groups.

## Material and Methods

### Selection of animals

One hundred pigs classified as *S*. *aureus* carriers (n = 44) and non-carriers (n = 56) were selected from an original population of 600 pigs sampled between May and October 2013 in two previous studies in Denmark [15, 16]. All pigs originated from 20 Danish production farms from the Central Jutland Region and all farms except one had integrated production purchasing 1,000 to 2,000 30-kg pigs per production cycle. Pigs were sampled during the last three weeks of the production cycle and farmers did not report antimicrobial treatments during this time. Non-carriers were included only if they originated from farms with at least one persistent carrier (one pig positive to *S*. *aureus* in three consecutive samplings). In addition to this inclusion criterion, the selection of pigs was based on the value of a pig random effect (RE) calculated in the previous quantitative longitudinal study [15]. Each pig from the sampled population was assigned a RE which takes values between 1 and -1. This value represents how much of the carriage status of a pig is due to individual factors and not environmental ones. The RE was estimated from the logistic regression model used in the previous study [15], which took into account i) the number of times a pig was positive, ii) the nasal counts (CFU/swab) recorded at each sampling time, and iii) the level of *S*. *aureus* exposure at the pen and the farm level (this one estimated by the number of carriers in the same pen and farm respectively, and their corresponding nasal counts) [15]. The resulting RE sorted the entire population of pigs, in a way that a pig with RE = 1 represented the individual with the highest CFU/swab, in all three sampling points and with no other carriers in the same pen or farm, whereas a pig with RE = -1 represented a negative pig in all three sampling points surrounded only by pigs carrying the highest CFU/swab. The final selection of 100 pigs was designed to include only those with RE values closer to 1 or -1, therefore selecting pigs where *S*. *aureus* carriage had the strongest individual (and not environmental) component, and excluding pigs with RE values close to 0, whose carriage phenotype may respond to environmental load. Data on the genotype of pigs were included in the study to identify possible differential features of the microbiome in pigs displaying the AA genotype, previously associated with non-carriage [16]. These individuals were compared to individuals with the GG (carriers) and AG (heterozygotes not associated with either of the two phenotypes) genotypes. Seventy-four of the 100 pigs included in the study were previously genotyped [16]. The remaining 26 pigs were genotyped for the same genetic marker using a TaqMan^®^ assay (Applied Biosystems, Foster City, USA) targeting the single nucleotide polymorphism (MARC0099960) correlated with *S*. *aureus* carriage (genotype GG) or non-carriage (genotype AA) [16]. Genotyping was performed according to manufacturer's recommendation on a Stratagene MX3005P qPCR System (Agilent Technologies, Santa Clara, CA, USA) including one control animal for each genotype (AA, AG, GG) from the previous sequenced collection of pigs.

### Nasal microbiome analysis

Total nucleic acid was extracted from nasal swabs (E-Swab, Copan Diagnostics Inc., USA) using the automated system QIASymphony^®^ SP and the QIASymphony DSP Virus/Pathogen MiniKit, v.1 (QIAGEN, Germany). The V3-V5 region of the 16S rRNA gene was amplified using universal forward primer 341F (5′-CCTACGGGNGGCWGCAG-3′) and the reverse primer 926R (5´-CCGTCAATTCMTTTRAGT-3´). Uni-directional sequencing was performed on a 454 GS FLX titanium system (454 Life Sciences, USA) at BGI (Shenzhen, China) as previously described [17]. Raw data analysis was performed in the BION meta package (Danish Genome Institute, Denmark) [18]. The workflow recipe is provided in S1 File. In brief, the workflow consisted of an initial de-multiplexing of sequences according to the primer and barcodes followed by removal of primer remnants from both ends as well as end regions with base quality less than 96%. Minimum sequence length was 180bp and it was required that at least 90% of all bases were of 95% quality or better. Identical sequences were merged, while preserving original read counts. Next, sequences were chimera-checked with default stringency. Non-chimeric sequences were matched against the Ribosomal Database Project (RDP) 11.03 [19] using a subset of sequences that comprised the entire length of the targeted amplicon. Sequence similarity required a minimum of 95% of matched bases. Output similarities were then mapped onto the RDP taxonomy [20] and abundance tables were generated for all levels from phylum to species. Data analysis was performed in R version 3.2.2 [21]. The generated matrix with raw read counts was analysed using the DESeq2 package version 1.10.0 [22] which uses shrinkage estimators, fold change values and controls false discovery rate by calculating adjusted p-Values. We investigated OTUs with different abundance between *S*. *aureus* carriers and non-carriers using two classification system of the pigs i) phenotypic classification as *S*. *aureus* carriers (CS = 1) and non-carriers (CS = 0) and ii) genotypic classification (MARC0099960) as non-carrier-associated genotype (AA) and the other two genotypes (AG or GG). It should be noted that genotypes AG and GG were grouped in order to identify bacterial species of interest in the AA genotype, as this is the genotype potentially colonized by *S*. *aureus* antagonists. The ReportingTools package [23] was used to generate an interactive HTML reports listing the significant results obtained by DESeq2. In order to visualize differential abundance, count data were normalized by the variance-stabilizing transformation (VST) as recommended by others [22, 24]. A heat map was generated by pheatmap 1.0.7 [25] using this transformed count data (VST).

## Results

### Characteristics of the pigs included in the study

The phenotypic and genotypic characteristics of the 100 pigs included in this study are shown in Table 1. Genotyping using the TaqMan^®^ Real-Time PCR assay was successful in a total of 23 animals. The signal did not clearly designate the genotypes of three animals, which were excluded from the analysis of differential abundance between *S*. *aureus* carriage genotypes.

**Table 1:** Number of pigs included in the study and their phenotypic (carriage status) and genotypic (MARC0099960) characterization in relation to *Staphylococcus aureus* carriage.

		Genotype			
No. of pigs	Carriage status (CS)	AA	AG	GG	NT ^a^
56	CS = 0	25	19	10	2
44	CS = 1	6	25	12	1

^a^ NT, non-typeable by TaqMan^®^ Real-Time PCR assay.

### 16S DNA sequencing and analysis of differential abundance

Sequencing, quality filtering and mapping resulted in 701,304 mapped V3-V5-region sequences, ranging between 1,686–46,096 copies per sample (average 7,013, SD = 6,325), which corresponded to 296 operational taxonomic units (OTUs). The workflow followed in this study (S1 File) was able to identify 164 of the 296 OTUs (55%) at the species level, while the remaining OTUs mapped to unclassified genera (34%) or upper taxonomic groups (11%). Seven phyla were identified, being *Proteobacteria* the most abundant (46% of sequences) and *Firmicutes* the most diverse (51% of all identified OTUs). Abundance and diversity of all phyla are shown in Table 2.

**Table 2:** Abundance (% of V3-V5 16S rRNA gene sequences) and diversity (% of OTUs) of the seven bacterial phyla identified in the pig nasal microbiota.

Phylum	Abundance (%)	Diversity (%)
*Proteobacteria*	46	34
*Firmicutes*	33	51
*Bacteriodetes*	20	11
*Unclassified bacteria*	0.4	0.3
*Actinobacteria*	0.09	3
*Planctomycetes*	0.03	0.3
*Fusobacteria*	0.02	0.3

The complete output matrix from BION is provided in S2 File and the results of the DESeq2 analysis are provided in S3 and S4 Files. The list of 25 OTUs with significant different abundance (adjusted p-value<0.05) between the pig phenotypes (carriers vs non-carriers) and between the genotypes (AA vs AG or GG) identified in the DESeq2 analysis are shown in Tables 3 and 4, respectively. The list of significant OTUs is also shown in S5 and S6 Files including visualization by plots of differential abundance. Presence/absence of the OTUs significantly associated to carriers and non-carriers across farms and individuals is shown in S1 and S2 Figs. A scheme of the taxonomy of the identified significant OTUs is provided for guidance (S3 Fig).

**Table 3:** Statistical values and absolute abundance of the 22 operational taxonomic units (OTUs) with significant differential abundance between *Staphylococcus aureus* carriers and non-carriers. The degree of differential abundance is represented by log2 fold change (logFC) which indicates a positive or negative interaction (logFC >0 or <0) of the specified OTU with *S*. *aureus* carriage.

OTU	Abundance	logFC	p-Value	Adjusted p-Value
*Acinetobacter lwoffii*	932	-2.22	5.02e-04	1.06e-02
*Acinetobacter soli*	61	-2.84	3.67e-03	4.06e-02
*Anaerococcus lactolyticus*	71	-3.22	5.53e-04	1.07e-02
*Facklamia tabacinasalis*	1356	-1.78	2.52e-03	3.25e-02
*Kurthia gibsonii*	15752	-3.49	1.88e-08	2.19e-06
*Leuconostoc mesenteroides*	1657	-1.98	3.30e-03	4.03e-02
*Leuconostoc pseudomesenteroides*	1004	-2.38	2.45e-03	3.25e-02
*Moraxella boevrei*	865	-2.62	1.17e-04	4.54e-03
Unclassified *Acinetobacter*	28127	-1.63	2.48e-03	3.25e-02
Unclassified Aerococcaceae	96	-1.90	3.60e-03	4.06e-02
Unclassified *Anaerococcus*	687	-3.33	1.03e-08	2.19e-06
Unclassified Chitinophagaceae	48	-2.84	4.43e-03	4.67e-02
Unclassified *Facklamia*	1204	-1.47	4.82e-04	1.06e-02
Unclassified *Faecalibacterium*	655	-1.09	1.07e-03	1.92e-02
Unclassified *Helcococcus*	827	-2.73	2.45e-06	1.89e-04
Unclassified Lachnospiraceae ^a^	1006	-1.21	3.11e-04	1.03e-02
Unclassified *Oscillibacter*	2458	-1.30	3.70e-04	1.06e-02
Unclassified *Prevotella*	1959	-1.60	4.37e-04	1.06e-02
Unclassified *Roseburia*	134	-3.09	8.49e-06	4.93e-04
Unclassified *Vagococcus*	2552	1.56	1.24e-03	2.05e-02
Unclassified *Wautersiella*	106131	2.49	3.17e-05	1.47e-03
*Vagococcus fluvialis*	11099	1.54	2.40e-03	3.25e-02

^a^ OTU with significant higher abundance in pigs with the non-carrier (AA) genotype.

**Table 4:** Statistical values and absolute abundance of the 4 operational taxonomic units (OTUs) with significant differential abundance in pigs displaying the genotype associated with *Staphylococcus aureus* non-carriage (AA), as compared to the other two genotypes (AG or GG). The degree of differential abundance is represented by log2 fold change (logFC), which indicates a positive or negative interaction (logFC >0 or <0) of the specified OTU in pigs with the AG or GG genotypes.

OTU	Abundance	logFC	p-Value	Adjusted p-Value
*Pasteurella multocida*	3012	3.24	4.45e-04	0.02600
Unclassified *Klebsiella*	3412	3.37	6.53e-04	0.02860
Unclassified Lachnospiraceae ^a^	1006	-1.25	4.04e-04	0.02600
Unclassified Porphyromonadaceae	955	-2.69	6.11e-06	0.00107

^a^ OTU with significant higher abundance in non-carriers.

**Fig 1:**
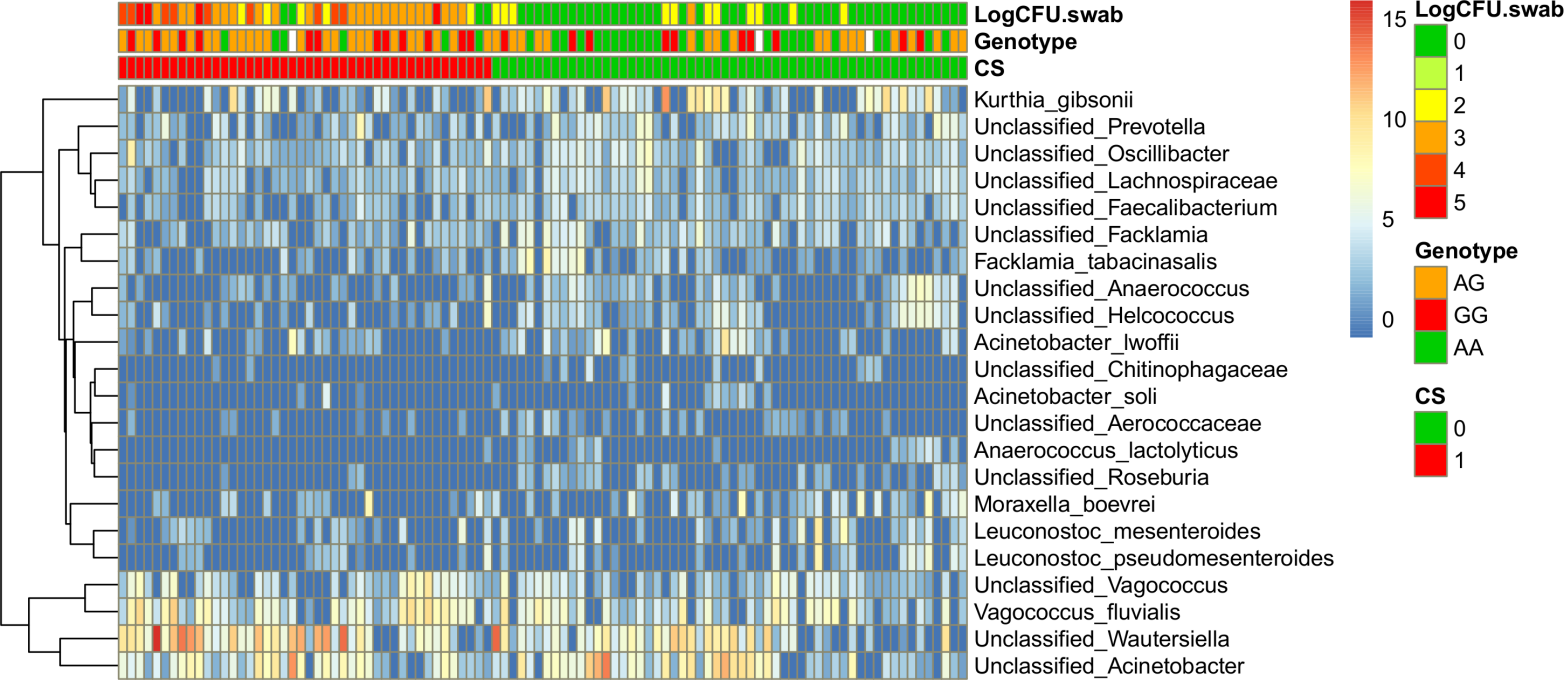
Heatmap of OTUs with significantly different abundance (adjusted *p*-values<0.05) between pigs *S*. *aureus* carriers and non-carriers according to their carriage status (CS = 1 and CS = 0 respectively). The genetic background (AA, AG and GG) and nasal loads in the first sampling point (LogCFU.swab) are also annotated. Values in the figure and legend correspond to the variance-stabilizing transformation (VST) of the original count data calculated with DESeq2.

Among the 22 OTUs differently abundant between the two pig phenotypes, 19 OTUs were associated with non-carriers (logFC<0) and three were associated with carriers (logFC>0) (Table 3 and S5 File). Among the four OTUs differently abundant in a specific pig host genotype, two opportunistic pathogenic organisms (*Pasteurella multocida* and *Klebsiella* spp.) were negatively correlated with the non-carrier-associated genotype AA (logFC<0) (Table 4 and S6 File). Only one of the two OTUs associated with this genotype, unclassified *Lachnospiraceae*, was also associated to the *S*. *aureus* non-carrier phenotype (Table 3). Heat maps visualizing the OTUs with significantly different abundance between the studied groups are shown in Fig 1 (VST normalization) alongside with annotated *S*. *aureus* nasal loads, carriage or CS and pig genotype.

## Discussion

This is the first study comparing the nasal microbiota of pigs classified as *S*. *aureus* carriers or non-carriers based on longitudinal and quantitative data and accounting for environmental exposure at the pen and farm levels. The only previous study investigating the relationship between *S*. *aureus* carriage and the pig nasal microbiome focused on MRSA, and classified a smaller number of animals (13 pigs from one farm) as carriers/non-carriers using a cross-sectional approach [26], which does not allow discrimination between truly colonized and contaminated pigs. The longitudinal quantitative culture-based approach used to classify *S*. *aureus* carriage in our study allows a higher positive predictive (i.e. a higher probability that subjects classified as carriers are truly carriers) according to the current standards for definition of *S*. *aureus* carriage in humans [27]. Another original aspect of the study was the inclusion of host genotypic data for all the pigs tested, enabling us to identify differential features of the microbiome in pigs displaying the AA genotype associated with non-carriage [16]. Furthermore, in order to identify the bacterial species differently abundant in the presence/absence of *S*. *aureus*, we used DESeq2, a method that has been shown to provide one of the best approaches to detect differentially abundant species compared to other tools commonly used in microbiome studies, such as rarefying or analysis of proportions, which underestimate uncertainty and fail to predict false positives [24]. Interestingly, this R package proposes methods for data transformation such as variance stabilizing transformation (VST), which we used to provide a sound visualization of our analysis [22].

Based on previous studies of nasopharyngeal microbiomes using the same sequencing platform, the total and average abundance/reads per sample was within standard values [28–30], or somewhat below [31]. The number of OTUs was moderately lower than in other nasal microbiomes [26, 31, 32], but still within the range of those found in the tonsils of pigs [28]. This is attributed by the authors to possible incomplete DNA recovery from nasal swabs by the automated DNA extraction system. Due to this limitation, a detailed ecological description of the pig nasal microbiome was omitted, and the analysis focused on differential abundance of the identified species, that we consider well represented as indicated by abundance data.

### Outline of the OTUs associated to *S*. *aureus* non-carriers

Analysis of the nasal microbiota of the 100 individuals included in the study led to identification of 20 OTUs associated with the non-carriage status (18 associated to the non-carrier phenotype, one associated to the non-carrier genotype AA, and one associated to both), eight of which were identified at the species level. Most of these OTUs belonged to Firmicutes but also to Bacteriodetes and Proteobacteria (S3 Fig). The majority of the identified OTUs are commonly found in bacterial communities in the upper respiratory tract of humans and animals, including order Clostridiales (family Lachnospiraceae), families Aerococcaceae and Porphyromonadaceae [33, 34], and genera such as *Anaerococcus* [35], *Prevotella* [36–38], *Acinetobacter* [34, 36] and *Moraxella* [35, 36]. On the other hand, some OTUs are unusual inhabitants of the upper respiratory tract. These include obligate anaerobic bacteria such as the Clostridia members *Faecalibacterium* and *Oscillibacter* [5, 39], which have however been reported among the most predominant taxa in the nasal microbiome of pigs [40]. The strictly aerobic metabolism of these bacteria does not fit with the typical aerobic environment in the nostrils, and their presence in this habitat is likely consequent to pig behaviour and contamination of the snout with faecal material or soil.

Three classes of Firmicutes were associated with non-carriage: Bacilli (six OTUs), Clostridia (four OTUs) and Tissierellia (three OTUs). Among the Lactobacillales, *Leuconostoc* spp. are non-pathogenic, lactic acid bacteria with known probiotic potential [41]. In particular, some *Leuconostoc mesenteroides* strains are known to produce bacteriocins able to inhibit the growth of food-borne pathogens and display adequate physicochemical probiotic properties for their application as bio-ingredients in raw and processed foods [42], making *Leuconostoc* an interesting candidate to study possible antagonism with *S*. *aureus*. This is not the case for *Facklamia* spp., which are considered opportunistic pathogens in humans and can display resistance to clinically important antimicrobials [43, 44]. The identified *F*. *tabacinasalis* is the only species considered non-pathogenic within the genus [45]. *Kurthia gibsonii* was the OTU with the strongest association to non-carriers (logFC = -3.49). However, its antimicrobial/probiotic properties seem unexplored and most reports refer to clinical specimens [46] with the exception of one study where *K*. *gibsonii* is proposed for agricultural use as a microbial pesticide [47].

Since Clostridia are obligate anaerobes, we presume that they are not actively growing in an aerobic environment such as the nasal cavity. The reasons for the associations of these four clostridial OTUs with non-carriage are unknown but might be related to the effects of other non-anaerobic bacteria derived from faecal or soil contamination. However, anaerobic bacteria do not seem to be suitable organisms for probiotic applications for *S*. *aureus* control in the nostrils and in the environment of pigs. Very little is known about the ecology of the two representatives of the class Tissierellia, *Anaerococcus* and *Helcococcus*. As clostridia, *Anaerococcus* spp. are obligate anaerobes and therefore not suitable candidates for probiotic applications against *S*. *aureus* colonization. *Helcococcus* spp. are known to occur on human skin and have been reported in human and veterinary clinical specimens [48].

Among the Bacteriodetes taxa associated with *S*. *aureus* non-carriers, the genus *Prevotella* has known probiotic effect in the gut of weaned pigs, increasing the levels of luminal IgA and body weight [5]. Members of the family Porphyromonadaceae are considered environmental bacteria and common colonizers of the gut of piglets [49]. Among the Proteobacteria, *Acinetobacter* spp. are known as environmental bacteria and human skin colonizers [50]; and *Moraxella* spp. are mostly commensals of mucosal surfaces, including the identified *Moraxella boevrei*, which was first isolated from the nasal mucosae of healthy goats [51].

While some of the OTUs associated with non-carriers showed abundances below 100 V3-V5 counts (*A*. *soli*, *A*. *lactolyticus* and unclassified Aerococcaceae and Chitiniphagaceae), most OTUs were present at very high numbers such as unclassified *Acinetobacter* (28,127 counts) and *Kurthia gibsonii* (15,752 counts) (Table 3). Hypothetically, presence at large amounts may increase the possible antagonist potential of these OTUs, if for example antagonism is due to competition for resources or attachment to substrate. However, the antagonist potential of less abundant OTUs should not be disregarded as it could be effective thanks to other mechanisms such as production of antimicrobial compounds.

### Relationship between the nasal microbiome, host genetic predisposition and *S*. *aureus* carriage

Although only one OTU was in common between the two analyses, among the 18 OTUs significantly associated with the non-carrier phenotype, ten had a similar trend (negative logFC) for the AA genotype associated with non-carriage (unclassified *Acinetobacter*, unclassified *Aerococcaceae*, unclassified *Chitinophagaceae*, unclassified *Facklamia*, *Facklamia tabicinasalis*, unclassified *Faecalibacterium*, *Kurthia gibsonii*, unclassified *Oscillibacter*, unclassified *Prevotella* and unclassified *Roseburia*) (S4 File). Similarly, the OTU significantly associated with the genotype AA, unclassified *Porphyromonadaceae*, showed a similar trend with the non-carrier phenotype (S3 File). This partial agreement between the two analyses is not surprising given the strong association between phenotype and genotype observed in the previous study [16]. However, we would have expected more OTUs in common between the two analyses. Host genetic predisposition to *S*. *aureus* colonization and microbiome composition seem to be poorly interrelated based on the results of this study and the significant OTUs associated to the non-carrier genotype (AA) suggest that the genotype has limited influence on the nasal microbiome traits differentiating carriers from non-carriers. Moreover, comparison of the microbiome between the homozygotes did not identify any significant OTUs associated to non-carriers (data not shown). These differences suggest the presence of multiple factors influencing *S*. *aureus* colonization which may also be of environmental nature, such as feed, health status of the farms, antimicrobial use and vaccinations, as previously suggested [35].

### Comparison with other microbiome-*S*. *aureus*/MRSA association studies in pigs and humans

The results of similar studies investigating the pig or human nasal microbiome in relation to *S*. *aureus* carriage are variable and sometimes contradictory. The OTUs associated to pig *S*. *aureus* non-carriers in our study differed from those suggested as indicators of MRSA-negative pigs in a previous study in Canada [26]. Two of the OTUs associated to MRSA-negative pigs in that study, *Neisseria* and *Lactobacillus*, had no significant adjusted p-values in our study and showed logFC values with no remarkable or with opposite trends among the 20 *Lactobacillus* spp. identified in our study (S3 File). On the other hand, the majority of taxa associated to the *S*. *aureus* non-carrier status (13/20) belonged to Firmicutes, in agreement with the Canadian study [26]. The results of both studies indicate limited similarities to analogous studies in humans. However, not all the human studies agree in the linkage between *S*. *aureus* carriage and nasal microbiota composition in humans. For example, two studies [35, 52] suggested that *Propionibacterium* spp. (*P*. *acnes* and *P*. *granulosum*) are negatively associated to *S*. *aureus*, whereas a third study reached the opposite conclusion [31]. Similarly, contradictory associations between *S*. *aureus* carriage and *S*. *epidermidis* have been reported in these three studies [35, 52, 53]. The apparent inconsistencies between different studies may be due to a variety of factors. In particular, differences between human and pig studies include possible interspecies variations in addition to different phenotype classification criteria, laboratory and bioinformatics methods. The latter factors can also be responsible for the differences observed between our study and the previous study on pig MRSA carriers by Weese et al. [26]. As explained in the beginning of the discussion, our study provides a substantial improvement in power and soundness compared to the previous one. Thus, we are not surprised by the lack of agreement between the two studies. This study provides a list of interesting probiotic candidates that can be evaluated by future studies to confirm possible antagonistic effects on *S*. *aureus*. Although we acknowledge the limited number of OTUs and the possible occurrence of other potential *S*. *aureus* antagonists that may have been undetected due to the DNA extraction method used, our data provide a good basis for future studies pursuing identification of probiotic organisms for control of *S*. *aureus*/MRSA in pigs.

## Conclusion

Despite all the limitations of an observational study, this is the most comprehensive study investigating the complex interaction between *S*. *aureus*, the pig nasal microbiome and the genetic background and environment of individual pigs. We found 20 bacterial candidates that were associated with non-carriage of *S*. *aureus* in the nasal cavity of pigs highly exposed to this bacterium at the pen and the farm level. Further research is warranted to isolate these bacteria, evaluate their antagonistic effect on *S*. *aureus* and their safety and favourable growth characteristics for development of probiotic products for MRSA control in pig farming.

## Supporting Information

S1 Fig Presence/absence of five operational taxonomic units (OTUs) significantly associated to *Staphylococcus aureus* carriage in pigs. Light grey bars show the number of pigs included in the study per farm (Farms 1–15). Each farm is represented by two bars indicating the *Staphylococcus aureus* carriage status of the pigs (carriers = 1, non-carriers = 0). Dark grey bars represent the number of pigs carrying each of OTUs indicated in the title of the plots.
(PDF)

S2 Fig Presence/absence of 20 operational taxonomic units (OTUs) significantly associated to *Staphylococcus aureus* non-carriage in pigs. Light grey bars show the number of pigs included in the study per farm (Farms 1–15). Each farm is represented by two bars indicating the *Staphylococcus aureus* carriage status of the pigs (carriers = 1, non-carriers = 0). Dark grey bars represent the number of pigs carrying each of OTUs indicated in the title of the plots.
(PDF)

S3 Fig Taxonomic scheme of the 20 operational taxonomic units (OTUs) significantly associated to *Staphylococcus aureus* non-carriage in pigs. (JPG)

S1 File Workflow recipe used in the analysis of the nasal microbiome of 100 pigs in the raw sequences generated by 454 GS FLX titanium system sequencing. (TXT)

S2 File Output matrix of abundance of bacterial species identified in the nasal microbiome of 100 pigs using BION meta. (XLSX)

S3 File Raw output of the analysis of differential abundance between *Staphylococcus aureus* carriers and non-carriers in a population of 100 pigs as performed by the DESeq2 package in R run on nasal 16S rDNA sequence count data. (CSV)

S4 File Raw output of the analysis of differential abundance between *Staphylococcus aureus* non-carrier-associated genotype (AA) and other genotypes (AG and GG) in a population of 97 pigs as performed by the DESeq2 package in R run on nasal 16S rDNA sequence count data. (CSV)

S5 File Statistical values and graphical representation of the 22 operational taxonomic units (OTUs) with significant higher abundance in *Staphylococcus aureus* non-carriers. The degree of differential abundance is represented by log2 fold change (logFC) which indicates a positive or negative interaction (logFC >0 or <0) of the specified OTU in presence of *Staphylococcus aureus*. Plots representing the abundance of each OTU in the population of *Staphylococcus aureus* carriers (1) and non-carriers (0), p-Values and adjusted p-Values are also provided.
(ZIP)

S6 File Statistical values and graphical representation of the 4 operational taxonomic units (OTUs) with significant higher abundance in the *Staphylococcus aureus* non-carrier-associated genotype (AA). The degree of differential abundance is represented by log2 fold change (logFC) which indicates a positive or negative interaction (logFC >0 or <0) of the specified OTU in pigs with genotypes different than the non-carrier-associated genotype (AA). Plots representing the abundance of each OTU in the population of pigs with the non-carrier (AA) or other genotypes (AG/GG), p-Values and adjusted p-Values are also provided.
(ZIP)
