# Supplementary material for: Differential Analysis of the Nasal Microbiome of Pig Carriers or Non-Carriers of Staphylococcus aureus
Source: PLoS One. 2016 Aug 10;11(8):e0160331. doi: 10.1371/journal.pone.0160331 (PMC4980049; doi:10.1371/journal.pone.0160331)
Supplement: S1 Fig — Light grey bars show the number of pigs included in the study per farm (Farms 1–15). Each farm is represented by two bars indicating the Staphylococcus aureus carriage status of the pigs (carriers = 1, non-carriers = 0). Dark grey bars represent the number of pigs carrying each of OTUs indicated in the title of the plots. (PDF) [file pone.0160331.s001.pdf]

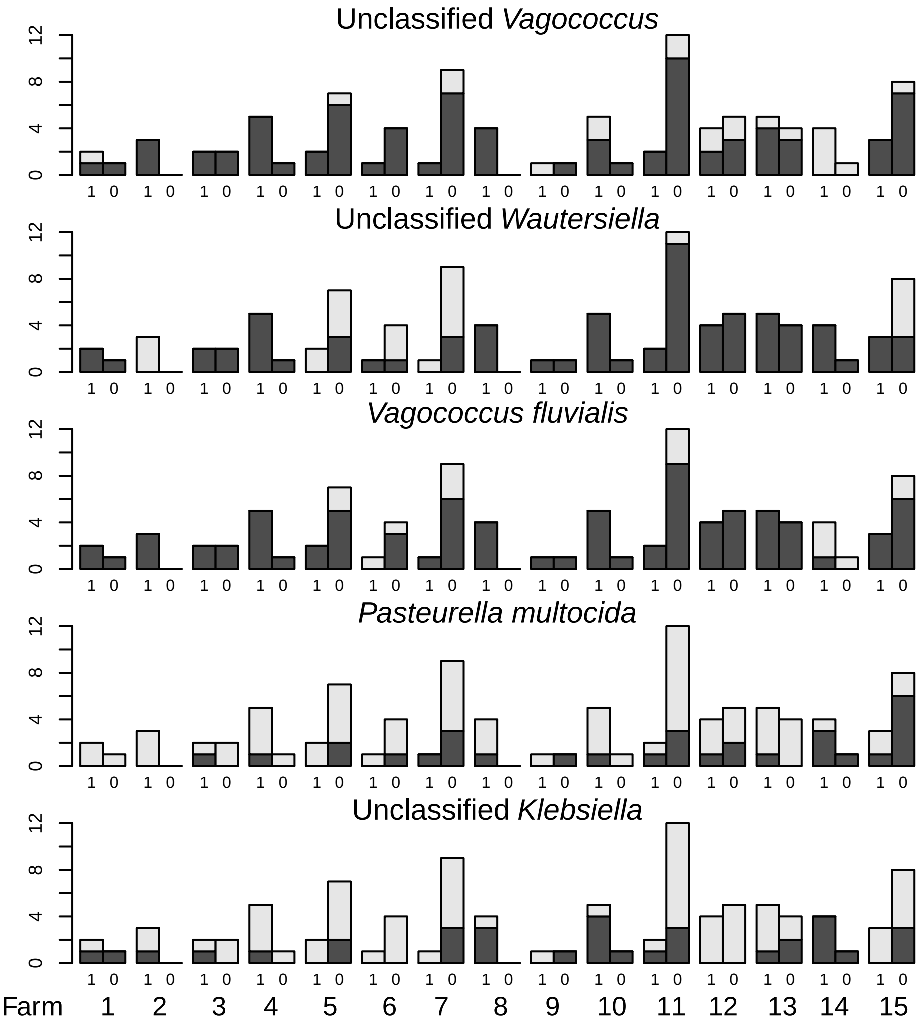
Unclassified Vagococcus
Unclassified Wautersiella
Vagococcus fluvialis
Pasteurella multocida
Unclassified Klebsiella
0
4
8
12
1 0
Farm
1 2 3 4 5 6 7 8 9 10 11 12 13 14 15
