# Supplementary figures and images for: Differential Analysis of the Nasal Microbiome of Pig Carriers or Non-Carriers of Staphylococcus aureus
Source: PLoS One. 2016 Aug 10;11(8):e0160331. doi: 10.1371/journal.pone.0160331 (PMC4980049; doi:10.1371/journal.pone.0160331)

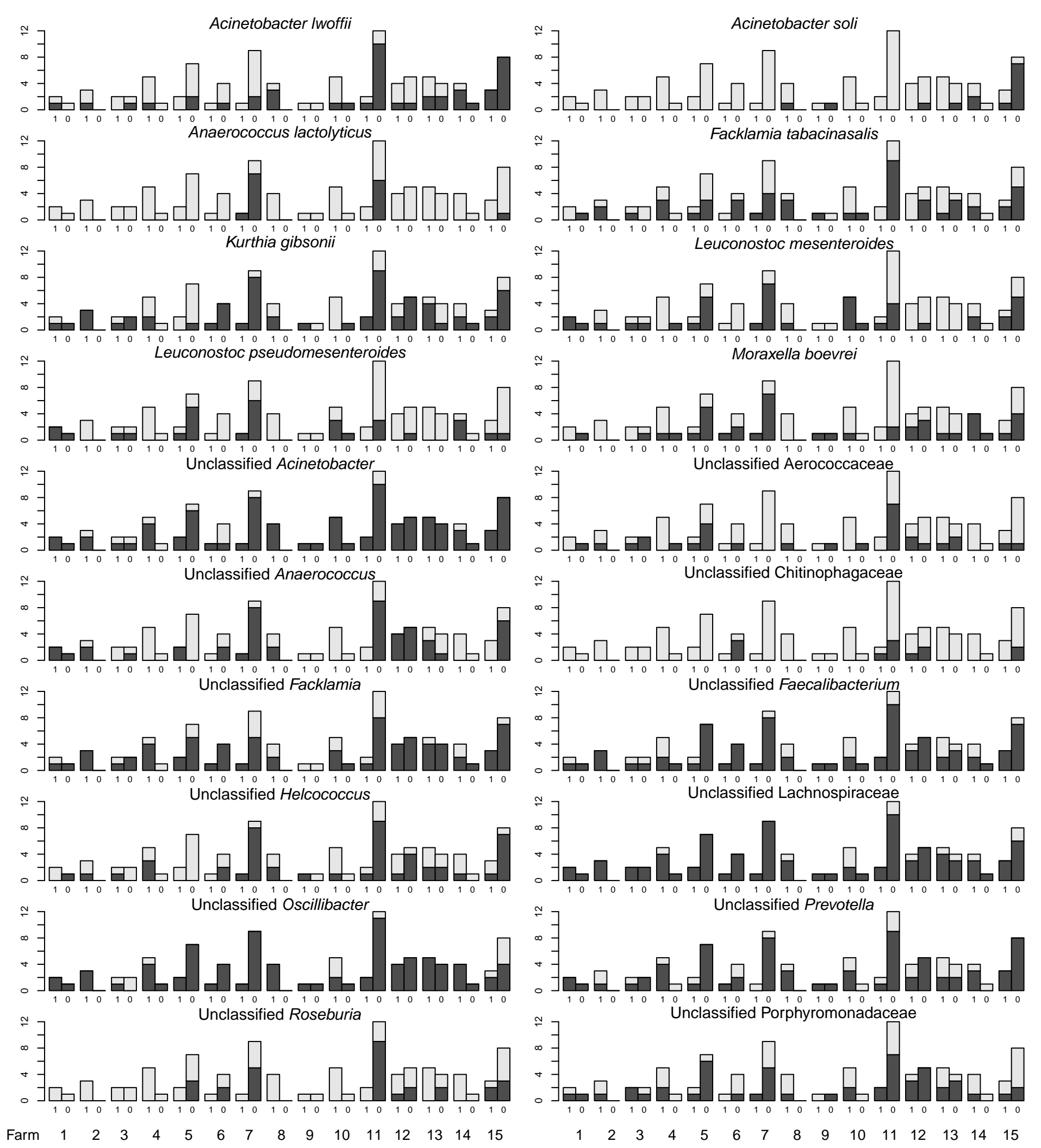

Supplement: S2 Fig — Light grey bars show the number of pigs included in the study per farm (Farms 1–15). Each farm is represented by two bars indicating the Staphylococcus aureus carriage status of the pigs (carriers = 1, non-carriers = 0). Dark grey bars represent the number of pigs carrying each of OTUs indicated in the title of the plots. (PDF) [file pone.0160331.s002.pdf]

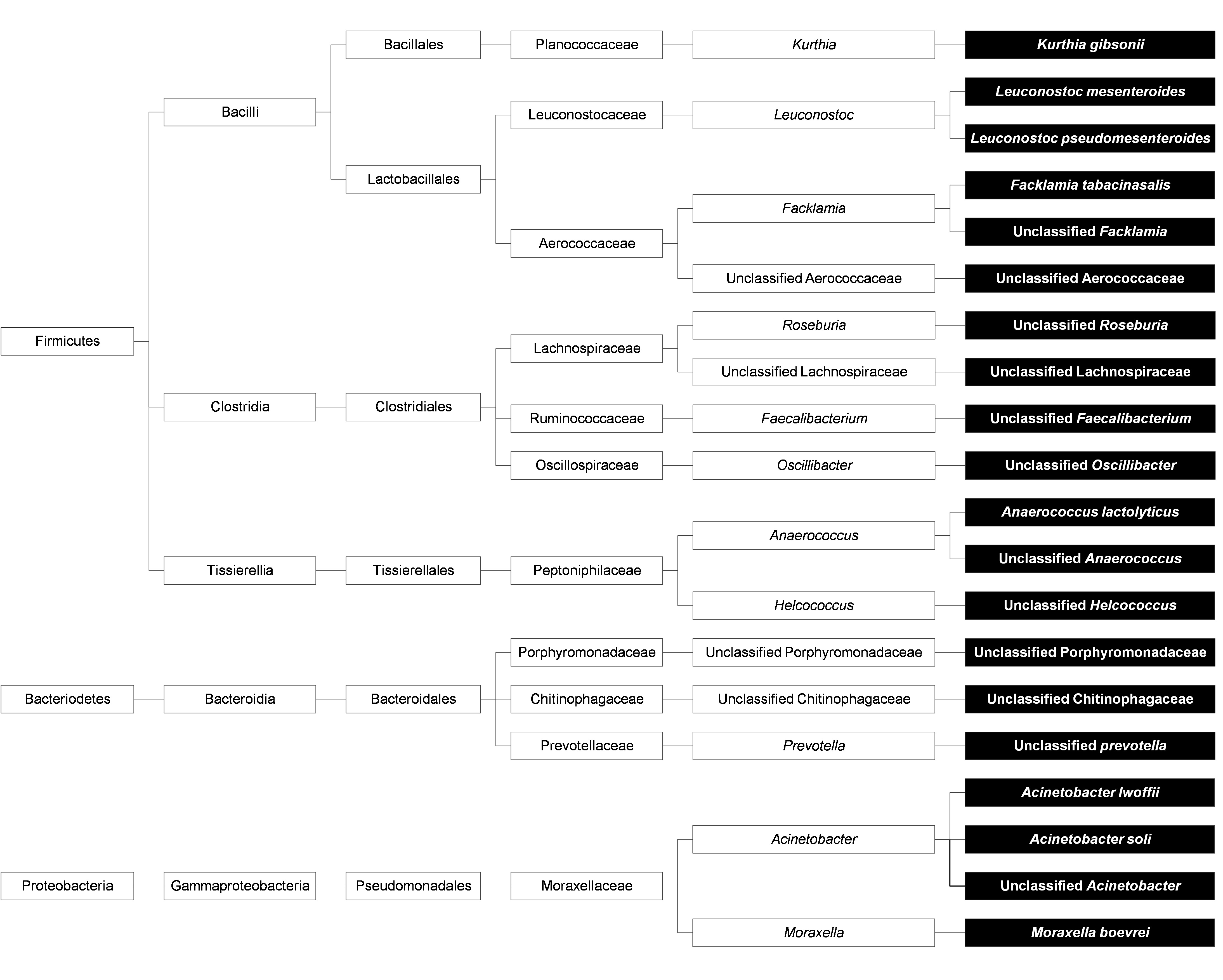

Supplement: S3 Fig — (JPG) [file pone.0160331.s003.jpg]

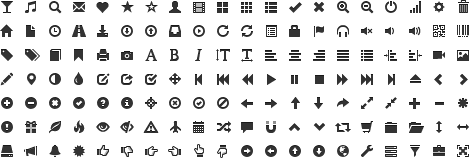

Supplement: S5 File — The degree of differential abundance is represented by log2 fold change (logFC) which indicates a positive or negative interaction (logFC >0 or <0) of the specified OTU in presence of Staphylococcus aureus. Plots representing the abundance of each OTU in the population of Staphylococcus aureus carriers (1) and non-carriers (0), p-Values and adjusted p-Values are also provided. (ZIP) [file pone.0160331.s008.zip › csslib/images/glyphicons-halflings.png]

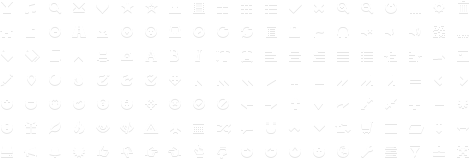

Supplement: S5 File — The degree of differential abundance is represented by log2 fold change (logFC) which indicates a positive or negative interaction (logFC >0 or <0) of the specified OTU in presence of Staphylococcus aureus. Plots representing the abundance of each OTU in the population of Staphylococcus aureus carriers (1) and non-carriers (0), p-Values and adjusted p-Values are also provided. (ZIP) [file pone.0160331.s008.zip › csslib/images/glyphicons-halflings-white.png]

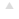

Supplement: S5 File — The degree of differential abundance is represented by log2 fold change (logFC) which indicates a positive or negative interaction (logFC >0 or <0) of the specified OTU in presence of Staphylococcus aureus. Plots representing the abundance of each OTU in the population of Staphylococcus aureus carriers (1) and non-carriers (0), p-Values and adjusted p-Values are also provided. (ZIP) [file pone.0160331.s008.zip › csslib/images/sort_asc.png]

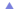

Supplement: S5 File — The degree of differential abundance is represented by log2 fold change (logFC) which indicates a positive or negative interaction (logFC >0 or <0) of the specified OTU in presence of Staphylococcus aureus. Plots representing the abundance of each OTU in the population of Staphylococcus aureus carriers (1) and non-carriers (0), p-Values and adjusted p-Values are also provided. (ZIP) [file pone.0160331.s008.zip › csslib/images/sort_asc_disabled.png]

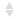

Supplement: S5 File — The degree of differential abundance is represented by log2 fold change (logFC) which indicates a positive or negative interaction (logFC >0 or <0) of the specified OTU in presence of Staphylococcus aureus. Plots representing the abundance of each OTU in the population of Staphylococcus aureus carriers (1) and non-carriers (0), p-Values and adjusted p-Values are also provided. (ZIP) [file pone.0160331.s008.zip › csslib/images/sort_both.png]

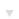

Supplement: S5 File — The degree of differential abundance is represented by log2 fold change (logFC) which indicates a positive or negative interaction (logFC >0 or <0) of the specified OTU in presence of Staphylococcus aureus. Plots representing the abundance of each OTU in the population of Staphylococcus aureus carriers (1) and non-carriers (0), p-Values and adjusted p-Values are also provided. (ZIP) [file pone.0160331.s008.zip › csslib/images/sort_desc.png]

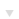

Supplement: S5 File — The degree of differential abundance is represented by log2 fold change (logFC) which indicates a positive or negative interaction (logFC >0 or <0) of the specified OTU in presence of Staphylococcus aureus. Plots representing the abundance of each OTU in the population of Staphylococcus aureus carriers (1) and non-carriers (0), p-Values and adjusted p-Values are also provided. (ZIP) [file pone.0160331.s008.zip › csslib/images/sort_desc_disabled.png]

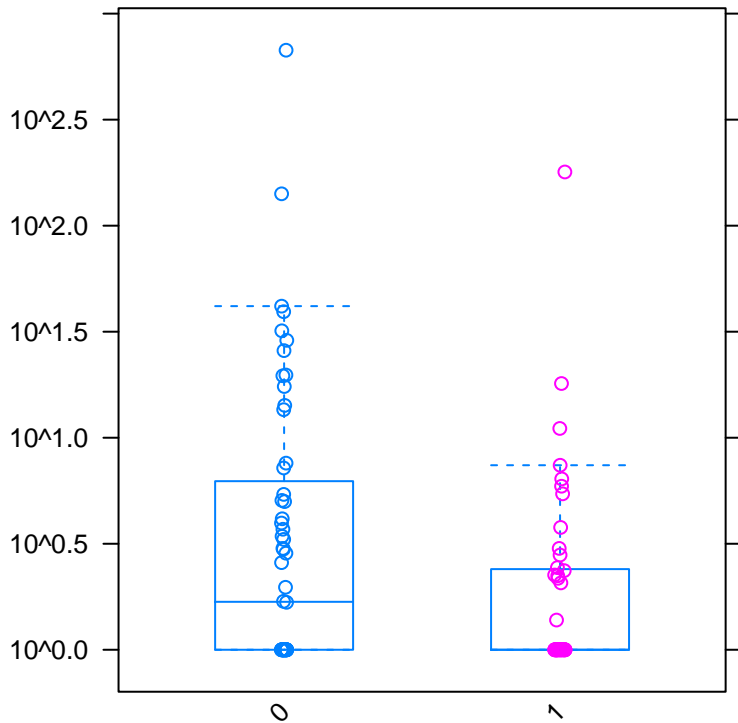

Supplement: S5 File — The degree of differential abundance is represented by log2 fold change (logFC) which indicates a positive or negative interaction (logFC >0 or <0) of the specified OTU in presence of Staphylococcus aureus. Plots representing the abundance of each OTU in the population of Staphylococcus aureus carriers (1) and non-carriers (0), p-Values and adjusted p-Values are also provided. (ZIP) [file pone.0160331.s008.zip › figuresDeSeq2 results design=Phenotype/boxplot.Acinetobacter_lwoffii.pdf]

Acinetobacter\_soli Normalized Counts

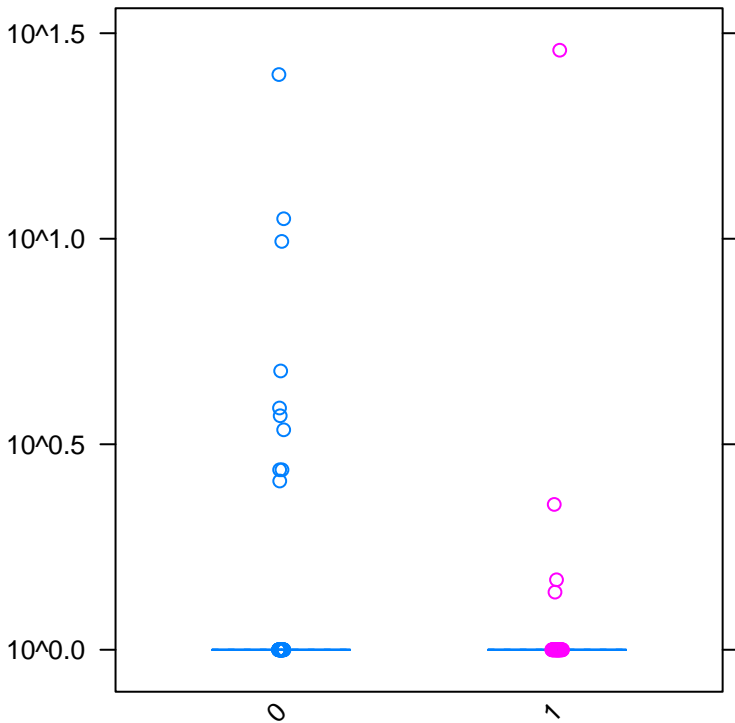

Supplement: S5 File — The degree of differential abundance is represented by log2 fold change (logFC) which indicates a positive or negative interaction (logFC >0 or <0) of the specified OTU in presence of Staphylococcus aureus. Plots representing the abundance of each OTU in the population of Staphylococcus aureus carriers (1) and non-carriers (0), p-Values and adjusted p-Values are also provided. (ZIP) [file pone.0160331.s008.zip › figuresDeSeq2 results design=Phenotype/boxplot.Acinetobacter_soli.pdf]

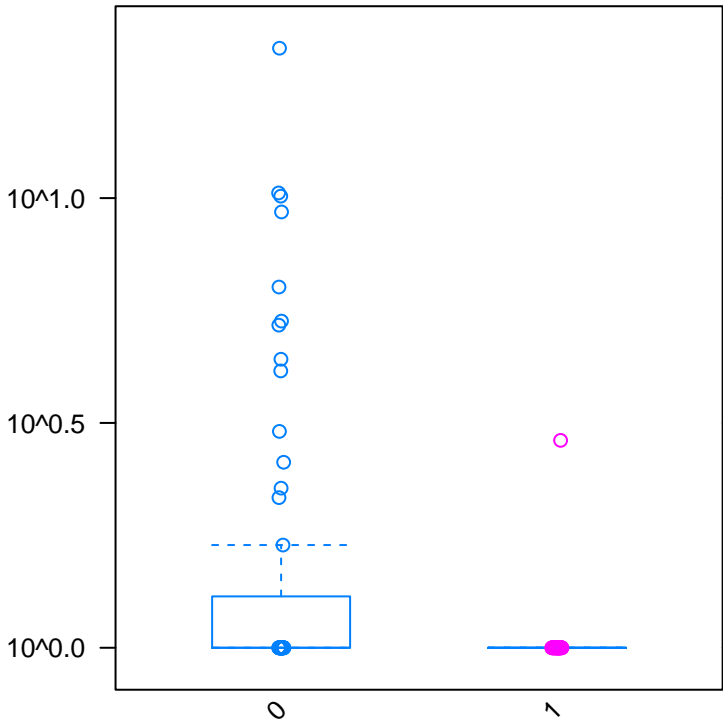

Supplement: S5 File — The degree of differential abundance is represented by log2 fold change (logFC) which indicates a positive or negative interaction (logFC >0 or <0) of the specified OTU in presence of Staphylococcus aureus. Plots representing the abundance of each OTU in the population of Staphylococcus aureus carriers (1) and non-carriers (0), p-Values and adjusted p-Values are also provided. (ZIP) [file pone.0160331.s008.zip › figuresDeSeq2 results design=Phenotype/boxplot.Anaerococcus_lactolyticus.pdf]

Facklamia\_tabacinasalis Normalized Counts

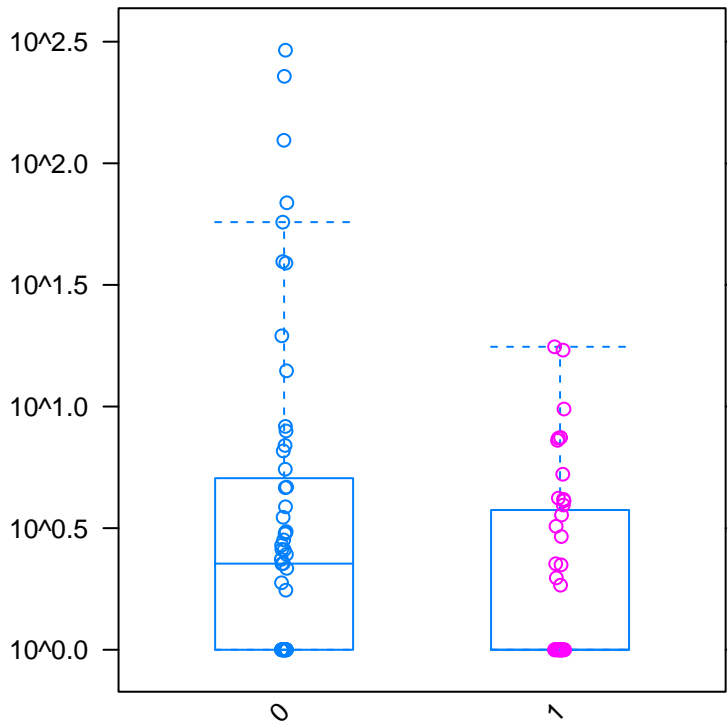

Supplement: S5 File — The degree of differential abundance is represented by log2 fold change (logFC) which indicates a positive or negative interaction (logFC >0 or <0) of the specified OTU in presence of Staphylococcus aureus. Plots representing the abundance of each OTU in the population of Staphylococcus aureus carriers (1) and non-carriers (0), p-Values and adjusted p-Values are also provided. (ZIP) [file pone.0160331.s008.zip › figuresDeSeq2 results design=Phenotype/boxplot.Facklamia_tabacinasalis.pdf]

Kurthia\_gibsonii Normalized Counts

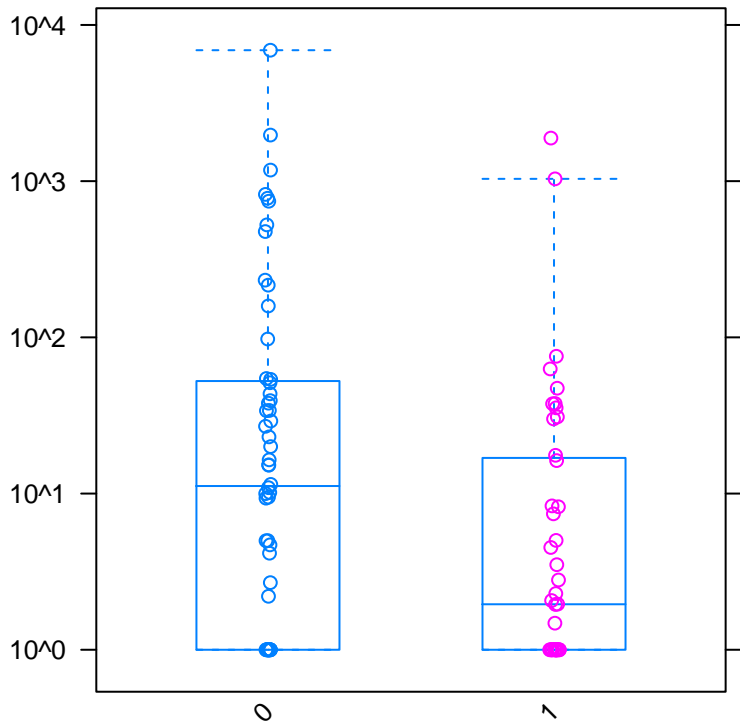

Supplement: S5 File — The degree of differential abundance is represented by log2 fold change (logFC) which indicates a positive or negative interaction (logFC >0 or <0) of the specified OTU in presence of Staphylococcus aureus. Plots representing the abundance of each OTU in the population of Staphylococcus aureus carriers (1) and non-carriers (0), p-Values and adjusted p-Values are also provided. (ZIP) [file pone.0160331.s008.zip › figuresDeSeq2 results design=Phenotype/boxplot.Kurthia_gibsonii.pdf]

Leuconostoc\_mesenteroides Normalized Counts

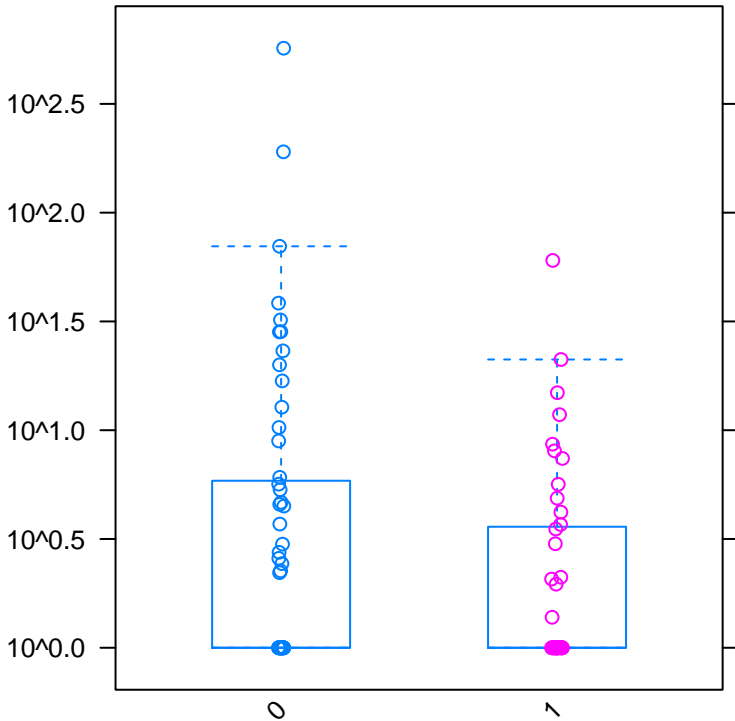

Supplement: S5 File — The degree of differential abundance is represented by log2 fold change (logFC) which indicates a positive or negative interaction (logFC >0 or <0) of the specified OTU in presence of Staphylococcus aureus. Plots representing the abundance of each OTU in the population of Staphylococcus aureus carriers (1) and non-carriers (0), p-Values and adjusted p-Values are also provided. (ZIP) [file pone.0160331.s008.zip › figuresDeSeq2 results design=Phenotype/boxplot.Leuconostoc_mesenteroides.pdf]

Leuconostoc\_pseudomesenteroides Normalized Counts

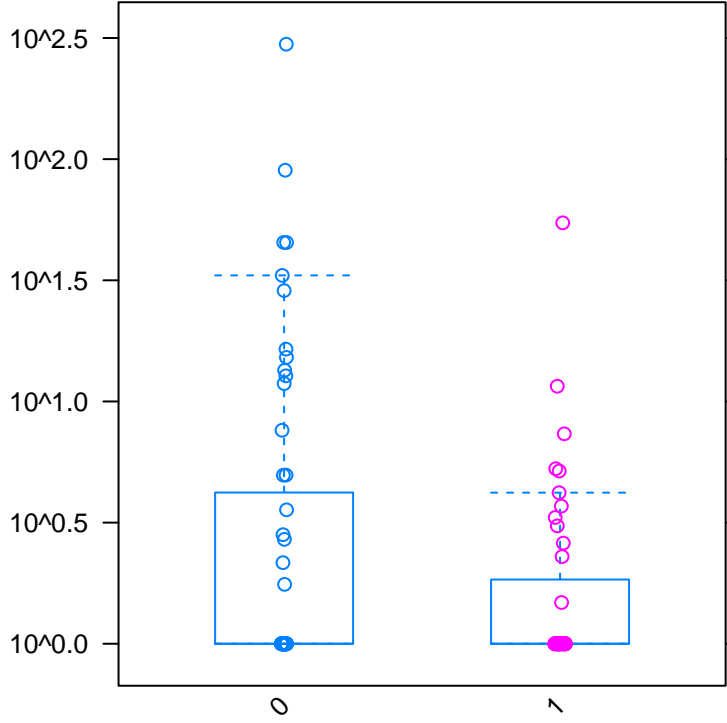

Supplement: S5 File — The degree of differential abundance is represented by log2 fold change (logFC) which indicates a positive or negative interaction (logFC >0 or <0) of the specified OTU in presence of Staphylococcus aureus. Plots representing the abundance of each OTU in the population of Staphylococcus aureus carriers (1) and non-carriers (0), p-Values and adjusted p-Values are also provided. (ZIP) [file pone.0160331.s008.zip › figuresDeSeq2 results design=Phenotype/boxplot.Leuconostoc_pseudomesenteroides.pdf]

Moraxella\_boevrei Normalized Counts

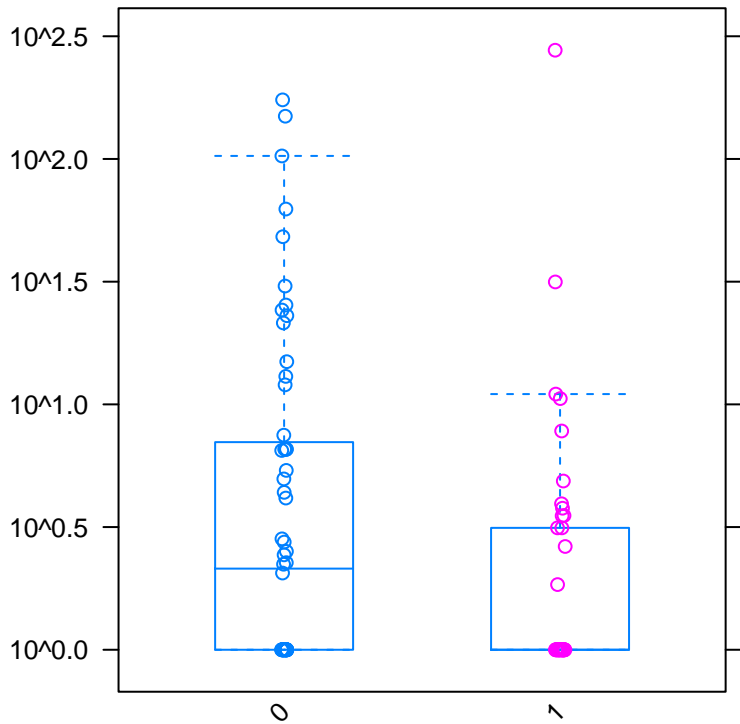

Supplement: S5 File — The degree of differential abundance is represented by log2 fold change (logFC) which indicates a positive or negative interaction (logFC >0 or <0) of the specified OTU in presence of Staphylococcus aureus. Plots representing the abundance of each OTU in the population of Staphylococcus aureus carriers (1) and non-carriers (0), p-Values and adjusted p-Values are also provided. (ZIP) [file pone.0160331.s008.zip › figuresDeSeq2 results design=Phenotype/boxplot.Moraxella_boevrei.pdf]

Unclassified\_Acinetobacter Normalized Counts

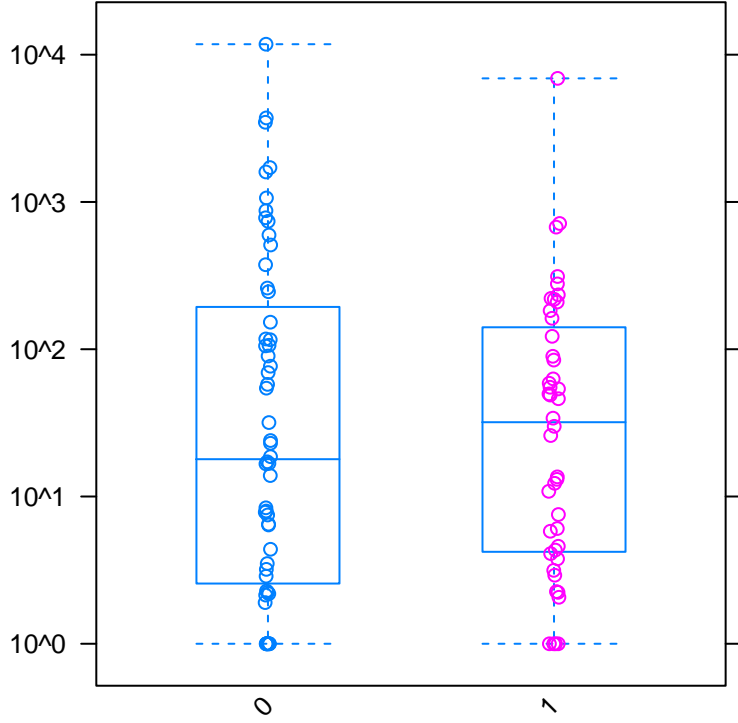

Supplement: S5 File — The degree of differential abundance is represented by log2 fold change (logFC) which indicates a positive or negative interaction (logFC >0 or <0) of the specified OTU in presence of Staphylococcus aureus. Plots representing the abundance of each OTU in the population of Staphylococcus aureus carriers (1) and non-carriers (0), p-Values and adjusted p-Values are also provided. (ZIP) [file pone.0160331.s008.zip › figuresDeSeq2 results design=Phenotype/boxplot.Unclassified_Acinetobacter.pdf]

Unclassified\_Aerococcaceae Normalized Counts

$10^{1.2}$   
 $10^{1.0}$   
 $10^{0.8}$   
 $10^{0.6}$   
 $10^{0.4}$   
 $10^{0.2}$   
 $10^{0.0}$

0

1

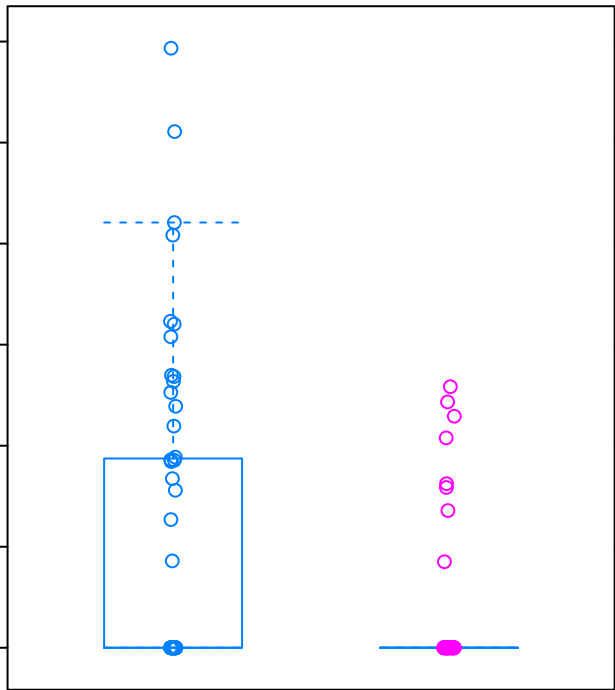

Supplement: S5 File — The degree of differential abundance is represented by log2 fold change (logFC) which indicates a positive or negative interaction (logFC >0 or <0) of the specified OTU in presence of Staphylococcus aureus. Plots representing the abundance of each OTU in the population of Staphylococcus aureus carriers (1) and non-carriers (0), p-Values and adjusted p-Values are also provided. (ZIP) [file pone.0160331.s008.zip › figuresDeSeq2 results design=Phenotype/boxplot.Unclassified_Aerococcaceae.pdf]

Unclassified\_Anaerococcus Normalized Counts

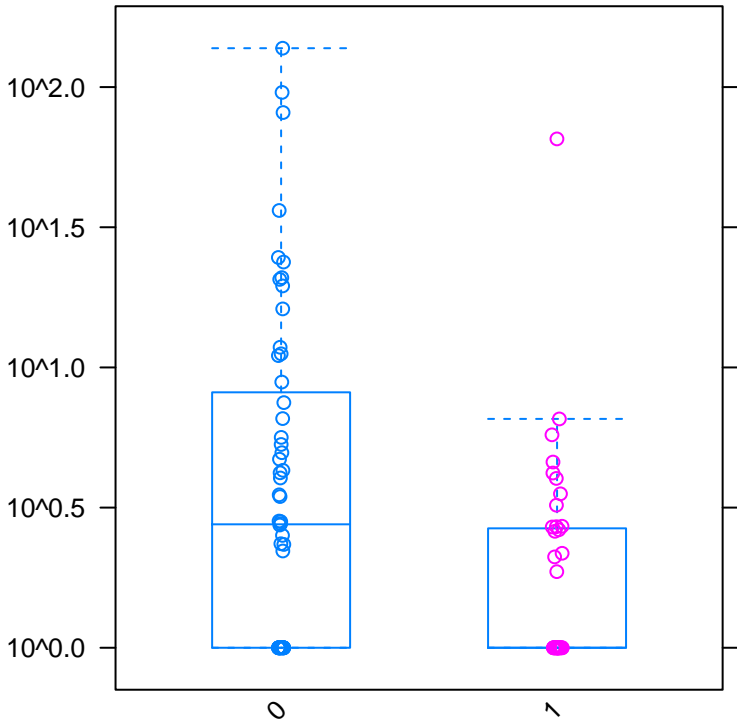

Supplement: S5 File — The degree of differential abundance is represented by log2 fold change (logFC) which indicates a positive or negative interaction (logFC >0 or <0) of the specified OTU in presence of Staphylococcus aureus. Plots representing the abundance of each OTU in the population of Staphylococcus aureus carriers (1) and non-carriers (0), p-Values and adjusted p-Values are also provided. (ZIP) [file pone.0160331.s008.zip › figuresDeSeq2 results design=Phenotype/boxplot.Unclassified_Anaerococcus.pdf]

Unclassified\_Chitinophagaceae Normalized Counts

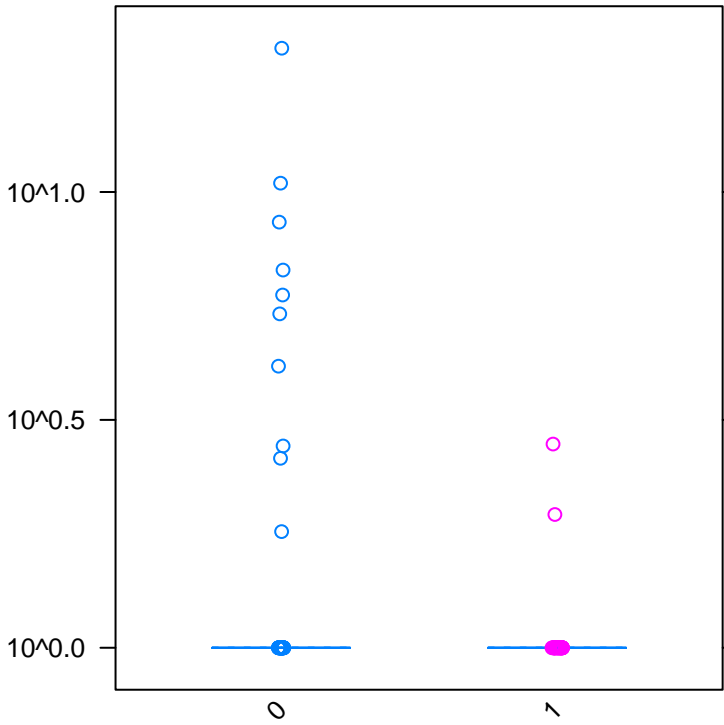

Supplement: S5 File — The degree of differential abundance is represented by log2 fold change (logFC) which indicates a positive or negative interaction (logFC >0 or <0) of the specified OTU in presence of Staphylococcus aureus. Plots representing the abundance of each OTU in the population of Staphylococcus aureus carriers (1) and non-carriers (0), p-Values and adjusted p-Values are also provided. (ZIP) [file pone.0160331.s008.zip › figuresDeSeq2 results design=Phenotype/boxplot.Unclassified_Chitinophagaceae.pdf]

Unclassified\_Facklamia Normalized Counts

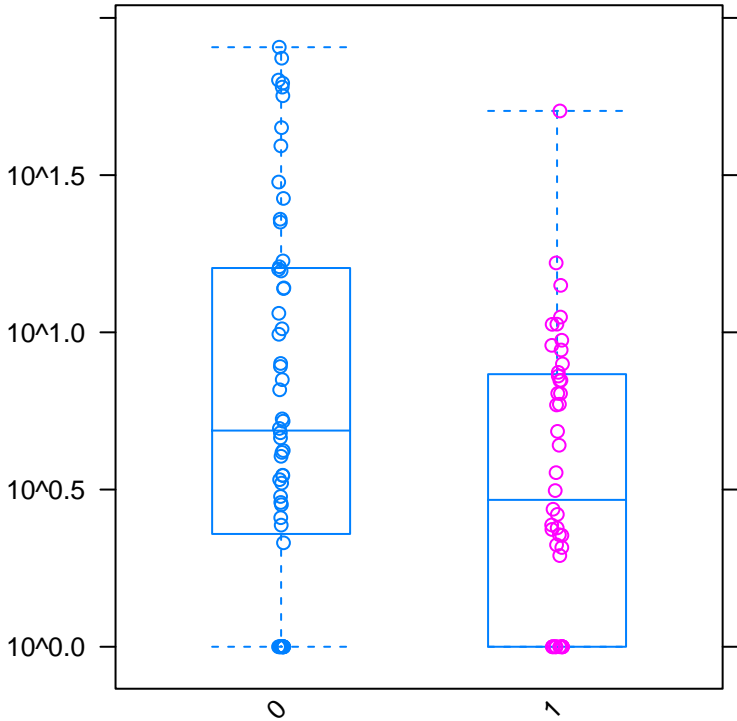

Supplement: S5 File — The degree of differential abundance is represented by log2 fold change (logFC) which indicates a positive or negative interaction (logFC >0 or <0) of the specified OTU in presence of Staphylococcus aureus. Plots representing the abundance of each OTU in the population of Staphylococcus aureus carriers (1) and non-carriers (0), p-Values and adjusted p-Values are also provided. (ZIP) [file pone.0160331.s008.zip › figuresDeSeq2 results design=Phenotype/boxplot.Unclassified_Facklamia.pdf]

Unclassified\_Faecalibacterium Normalized Counts

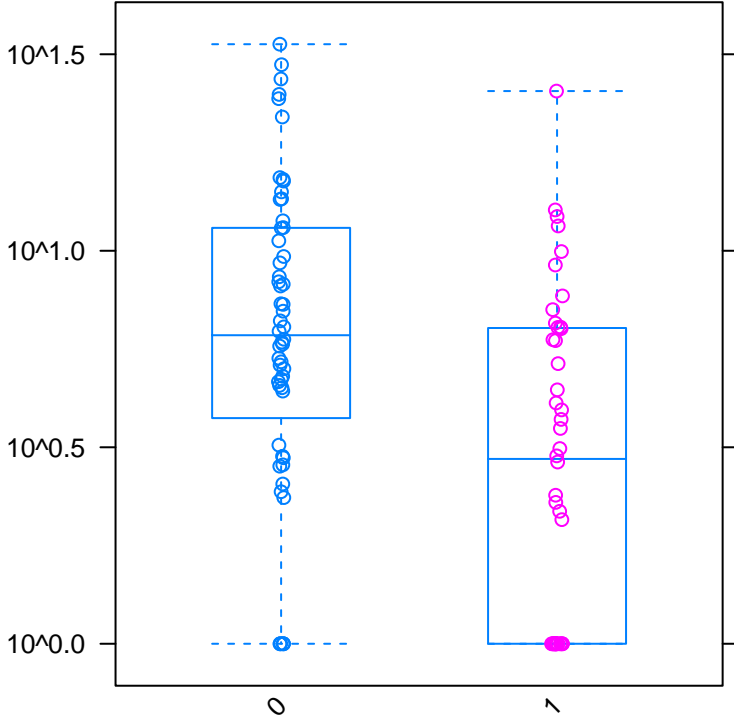

Supplement: S5 File — The degree of differential abundance is represented by log2 fold change (logFC) which indicates a positive or negative interaction (logFC >0 or <0) of the specified OTU in presence of Staphylococcus aureus. Plots representing the abundance of each OTU in the population of Staphylococcus aureus carriers (1) and non-carriers (0), p-Values and adjusted p-Values are also provided. (ZIP) [file pone.0160331.s008.zip › figuresDeSeq2 results design=Phenotype/boxplot.Unclassified_Faecalibacterium.pdf]

Unclassified\_Helcococcus Normalized Counts

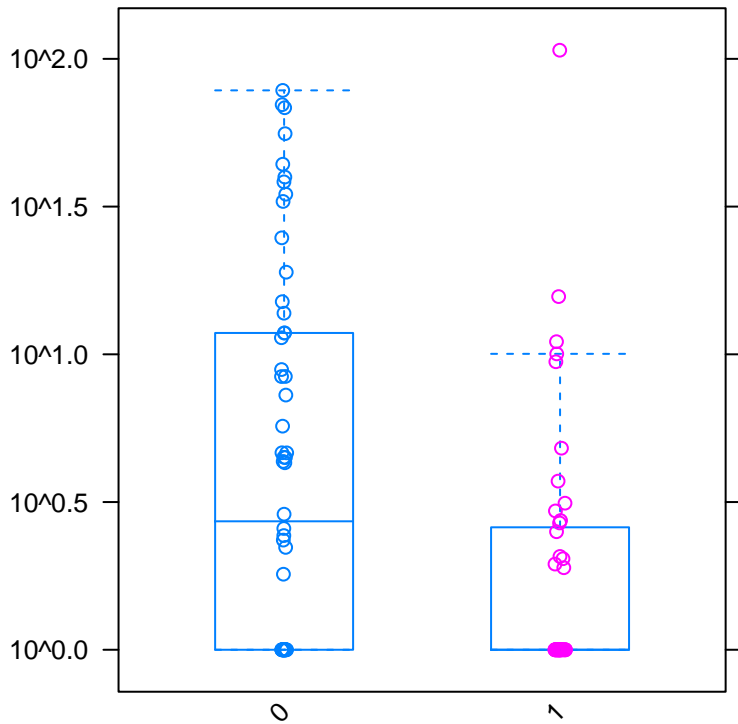

Supplement: S5 File — The degree of differential abundance is represented by log2 fold change (logFC) which indicates a positive or negative interaction (logFC >0 or <0) of the specified OTU in presence of Staphylococcus aureus. Plots representing the abundance of each OTU in the population of Staphylococcus aureus carriers (1) and non-carriers (0), p-Values and adjusted p-Values are also provided. (ZIP) [file pone.0160331.s008.zip › figuresDeSeq2 results design=Phenotype/boxplot.Unclassified_Helcococcus.pdf]

Unclassified\_Lachnospiraceae Normalized Counts

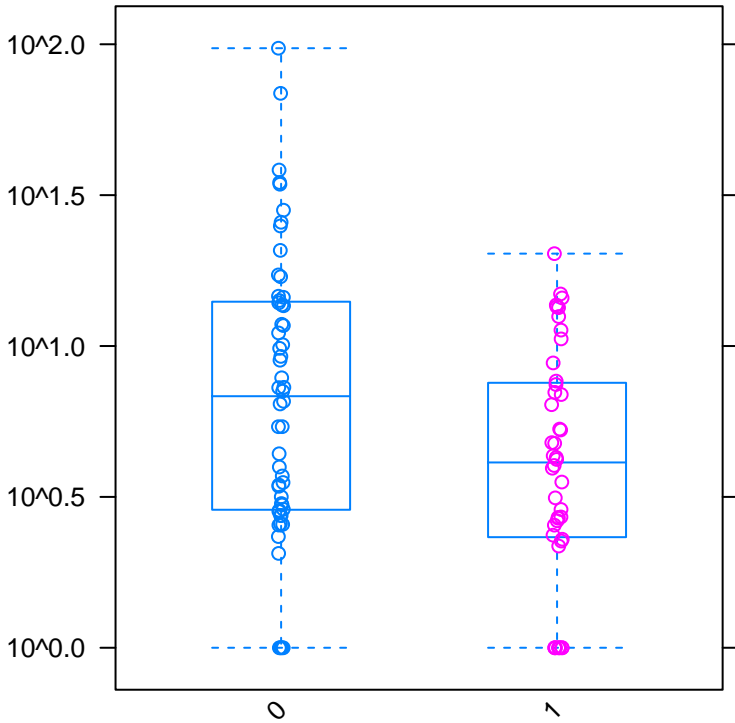

Supplement: S5 File — The degree of differential abundance is represented by log2 fold change (logFC) which indicates a positive or negative interaction (logFC >0 or <0) of the specified OTU in presence of Staphylococcus aureus. Plots representing the abundance of each OTU in the population of Staphylococcus aureus carriers (1) and non-carriers (0), p-Values and adjusted p-Values are also provided. (ZIP) [file pone.0160331.s008.zip › figuresDeSeq2 results design=Phenotype/boxplot.Unclassified_Lachnospiraceae.pdf]

Unclassified\_Oscillibacter Normalized Counts

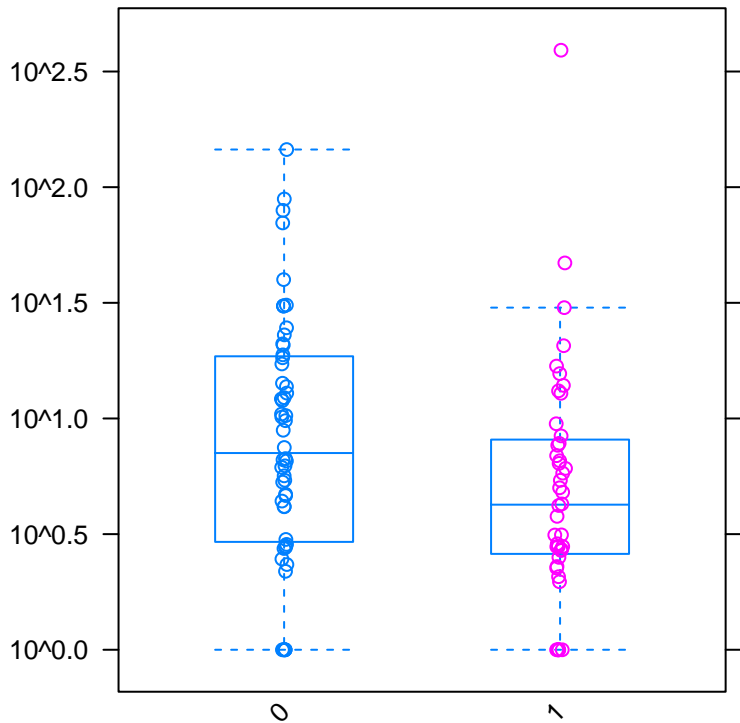

Supplement: S5 File — The degree of differential abundance is represented by log2 fold change (logFC) which indicates a positive or negative interaction (logFC >0 or <0) of the specified OTU in presence of Staphylococcus aureus. Plots representing the abundance of each OTU in the population of Staphylococcus aureus carriers (1) and non-carriers (0), p-Values and adjusted p-Values are also provided. (ZIP) [file pone.0160331.s008.zip › figuresDeSeq2 results design=Phenotype/boxplot.Unclassified_Oscillibacter.pdf]

Unclassified\_Prevotella Normalized Counts

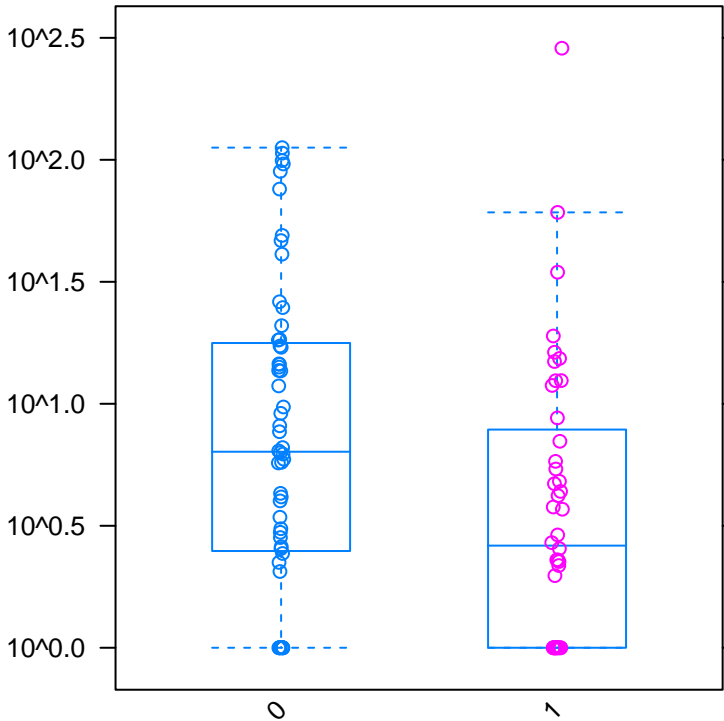

Supplement: S5 File — The degree of differential abundance is represented by log2 fold change (logFC) which indicates a positive or negative interaction (logFC >0 or <0) of the specified OTU in presence of Staphylococcus aureus. Plots representing the abundance of each OTU in the population of Staphylococcus aureus carriers (1) and non-carriers (0), p-Values and adjusted p-Values are also provided. (ZIP) [file pone.0160331.s008.zip › figuresDeSeq2 results design=Phenotype/boxplot.Unclassified_Prevotella.pdf]

Unclassified\_Roseburia Normalized Counts

$10^{1.2}$   
 $10^{1.0}$   
 $10^{0.8}$   
 $10^{0.6}$   
 $10^{0.4}$   
 $10^{0.2}$   
 $10^{0.0}$

0

1

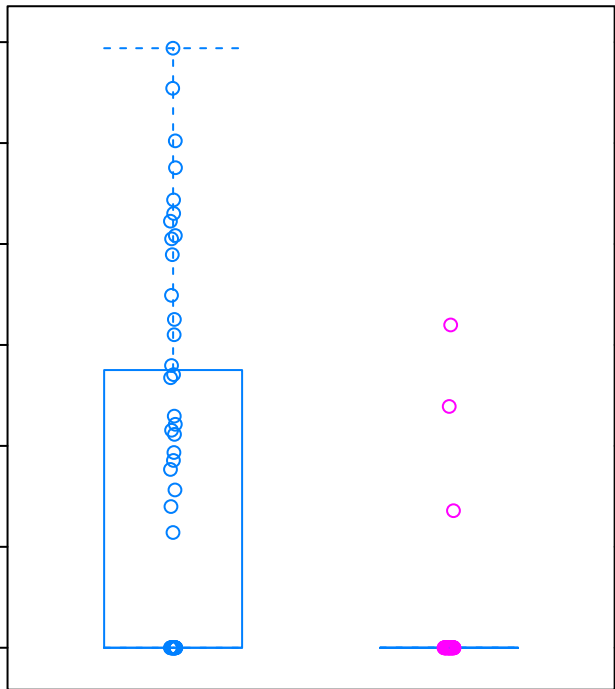

Supplement: S5 File — The degree of differential abundance is represented by log2 fold change (logFC) which indicates a positive or negative interaction (logFC >0 or <0) of the specified OTU in presence of Staphylococcus aureus. Plots representing the abundance of each OTU in the population of Staphylococcus aureus carriers (1) and non-carriers (0), p-Values and adjusted p-Values are also provided. (ZIP) [file pone.0160331.s008.zip › figuresDeSeq2 results design=Phenotype/boxplot.Unclassified_Roseburia.pdf]

Unclassified\_Vagococcus Normalized Counts

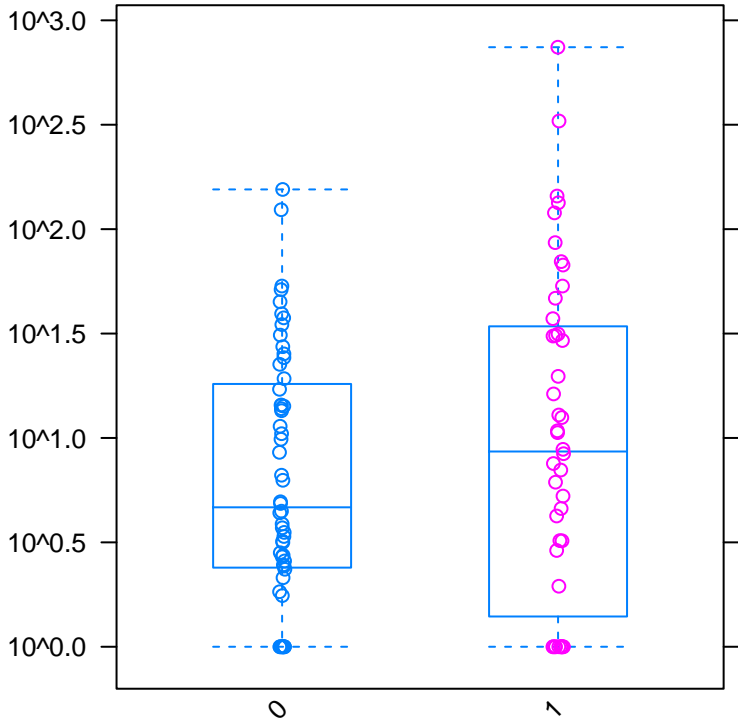

Supplement: S5 File — The degree of differential abundance is represented by log2 fold change (logFC) which indicates a positive or negative interaction (logFC >0 or <0) of the specified OTU in presence of Staphylococcus aureus. Plots representing the abundance of each OTU in the population of Staphylococcus aureus carriers (1) and non-carriers (0), p-Values and adjusted p-Values are also provided. (ZIP) [file pone.0160331.s008.zip › figuresDeSeq2 results design=Phenotype/boxplot.Unclassified_Vagococcus.pdf]

Unclassified\_Wautersiella Normalized Counts

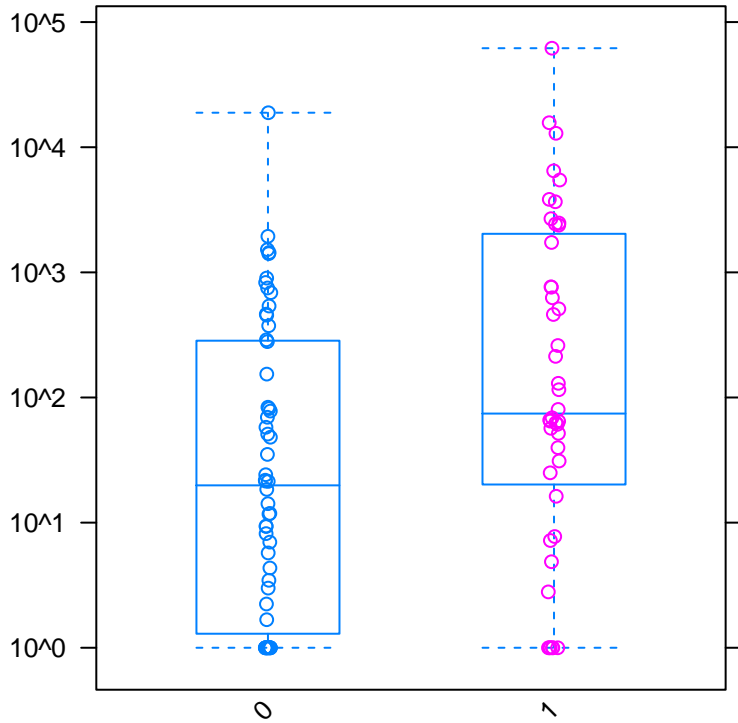

Supplement: S5 File — The degree of differential abundance is represented by log2 fold change (logFC) which indicates a positive or negative interaction (logFC >0 or <0) of the specified OTU in presence of Staphylococcus aureus. Plots representing the abundance of each OTU in the population of Staphylococcus aureus carriers (1) and non-carriers (0), p-Values and adjusted p-Values are also provided. (ZIP) [file pone.0160331.s008.zip › figuresDeSeq2 results design=Phenotype/boxplot.Unclassified_Wautersiella.pdf]

Vagococcus\_fluvialis Normalized Counts

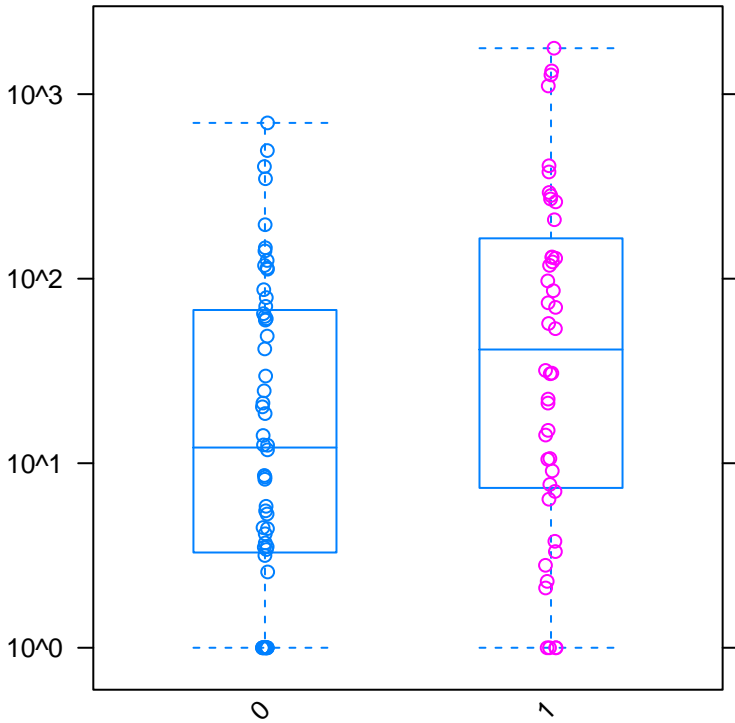

Supplement: S5 File — The degree of differential abundance is represented by log2 fold change (logFC) which indicates a positive or negative interaction (logFC >0 or <0) of the specified OTU in presence of Staphylococcus aureus. Plots representing the abundance of each OTU in the population of Staphylococcus aureus carriers (1) and non-carriers (0), p-Values and adjusted p-Values are also provided. (ZIP) [file pone.0160331.s008.zip › figuresDeSeq2 results design=Phenotype/boxplot.Vagococcus_fluvialis.pdf]

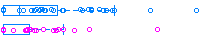

Supplement: S5 File — The degree of differential abundance is represented by log2 fold change (logFC) which indicates a positive or negative interaction (logFC >0 or <0) of the specified OTU in presence of Staphylococcus aureus. Plots representing the abundance of each OTU in the population of Staphylococcus aureus carriers (1) and non-carriers (0), p-Values and adjusted p-Values are also provided. (ZIP) [file pone.0160331.s008.zip › figuresDeSeq2 results design=Phenotype/mini.Acinetobacter_lwoffii.png]

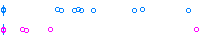

Supplement: S5 File — The degree of differential abundance is represented by log2 fold change (logFC) which indicates a positive or negative interaction (logFC >0 or <0) of the specified OTU in presence of Staphylococcus aureus. Plots representing the abundance of each OTU in the population of Staphylococcus aureus carriers (1) and non-carriers (0), p-Values and adjusted p-Values are also provided. (ZIP) [file pone.0160331.s008.zip › figuresDeSeq2 results design=Phenotype/mini.Acinetobacter_soli.png]

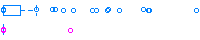

Supplement: S5 File — The degree of differential abundance is represented by log2 fold change (logFC) which indicates a positive or negative interaction (logFC >0 or <0) of the specified OTU in presence of Staphylococcus aureus. Plots representing the abundance of each OTU in the population of Staphylococcus aureus carriers (1) and non-carriers (0), p-Values and adjusted p-Values are also provided. (ZIP) [file pone.0160331.s008.zip › figuresDeSeq2 results design=Phenotype/mini.Anaerococcus_lactolyticus.png]

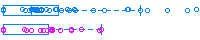

Supplement: S5 File — The degree of differential abundance is represented by log2 fold change (logFC) which indicates a positive or negative interaction (logFC >0 or <0) of the specified OTU in presence of Staphylococcus aureus. Plots representing the abundance of each OTU in the population of Staphylococcus aureus carriers (1) and non-carriers (0), p-Values and adjusted p-Values are also provided. (ZIP) [file pone.0160331.s008.zip › figuresDeSeq2 results design=Phenotype/mini.Facklamia_tabacinasalis.png]

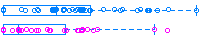

Supplement: S5 File — The degree of differential abundance is represented by log2 fold change (logFC) which indicates a positive or negative interaction (logFC >0 or <0) of the specified OTU in presence of Staphylococcus aureus. Plots representing the abundance of each OTU in the population of Staphylococcus aureus carriers (1) and non-carriers (0), p-Values and adjusted p-Values are also provided. (ZIP) [file pone.0160331.s008.zip › figuresDeSeq2 results design=Phenotype/mini.Kurthia_gibsonii.png]

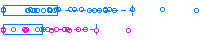

Supplement: S5 File — The degree of differential abundance is represented by log2 fold change (logFC) which indicates a positive or negative interaction (logFC >0 or <0) of the specified OTU in presence of Staphylococcus aureus. Plots representing the abundance of each OTU in the population of Staphylococcus aureus carriers (1) and non-carriers (0), p-Values and adjusted p-Values are also provided. (ZIP) [file pone.0160331.s008.zip › figuresDeSeq2 results design=Phenotype/mini.Leuconostoc_mesenteroides.png]

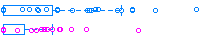

Supplement: S5 File — The degree of differential abundance is represented by log2 fold change (logFC) which indicates a positive or negative interaction (logFC >0 or <0) of the specified OTU in presence of Staphylococcus aureus. Plots representing the abundance of each OTU in the population of Staphylococcus aureus carriers (1) and non-carriers (0), p-Values and adjusted p-Values are also provided. (ZIP) [file pone.0160331.s008.zip › figuresDeSeq2 results design=Phenotype/mini.Leuconostoc_pseudomesenteroides.png]

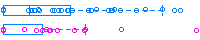

Supplement: S5 File — The degree of differential abundance is represented by log2 fold change (logFC) which indicates a positive or negative interaction (logFC >0 or <0) of the specified OTU in presence of Staphylococcus aureus. Plots representing the abundance of each OTU in the population of Staphylococcus aureus carriers (1) and non-carriers (0), p-Values and adjusted p-Values are also provided. (ZIP) [file pone.0160331.s008.zip › figuresDeSeq2 results design=Phenotype/mini.Moraxella_boevrei.png]

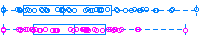

Supplement: S5 File — The degree of differential abundance is represented by log2 fold change (logFC) which indicates a positive or negative interaction (logFC >0 or <0) of the specified OTU in presence of Staphylococcus aureus. Plots representing the abundance of each OTU in the population of Staphylococcus aureus carriers (1) and non-carriers (0), p-Values and adjusted p-Values are also provided. (ZIP) [file pone.0160331.s008.zip › figuresDeSeq2 results design=Phenotype/mini.Unclassified_Acinetobacter.png]

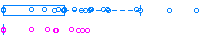

Supplement: S5 File — The degree of differential abundance is represented by log2 fold change (logFC) which indicates a positive or negative interaction (logFC >0 or <0) of the specified OTU in presence of Staphylococcus aureus. Plots representing the abundance of each OTU in the population of Staphylococcus aureus carriers (1) and non-carriers (0), p-Values and adjusted p-Values are also provided. (ZIP) [file pone.0160331.s008.zip › figuresDeSeq2 results design=Phenotype/mini.Unclassified_Aerococcaceae.png]

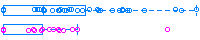

Supplement: S5 File — The degree of differential abundance is represented by log2 fold change (logFC) which indicates a positive or negative interaction (logFC >0 or <0) of the specified OTU in presence of Staphylococcus aureus. Plots representing the abundance of each OTU in the population of Staphylococcus aureus carriers (1) and non-carriers (0), p-Values and adjusted p-Values are also provided. (ZIP) [file pone.0160331.s008.zip › figuresDeSeq2 results design=Phenotype/mini.Unclassified_Anaerococcus.png]

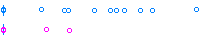

Supplement: S5 File — The degree of differential abundance is represented by log2 fold change (logFC) which indicates a positive or negative interaction (logFC >0 or <0) of the specified OTU in presence of Staphylococcus aureus. Plots representing the abundance of each OTU in the population of Staphylococcus aureus carriers (1) and non-carriers (0), p-Values and adjusted p-Values are also provided. (ZIP) [file pone.0160331.s008.zip › figuresDeSeq2 results design=Phenotype/mini.Unclassified_Chitinophagaceae.png]

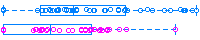

Supplement: S5 File — The degree of differential abundance is represented by log2 fold change (logFC) which indicates a positive or negative interaction (logFC >0 or <0) of the specified OTU in presence of Staphylococcus aureus. Plots representing the abundance of each OTU in the population of Staphylococcus aureus carriers (1) and non-carriers (0), p-Values and adjusted p-Values are also provided. (ZIP) [file pone.0160331.s008.zip › figuresDeSeq2 results design=Phenotype/mini.Unclassified_Facklamia.png]

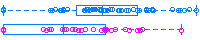

Supplement: S5 File — The degree of differential abundance is represented by log2 fold change (logFC) which indicates a positive or negative interaction (logFC >0 or <0) of the specified OTU in presence of Staphylococcus aureus. Plots representing the abundance of each OTU in the population of Staphylococcus aureus carriers (1) and non-carriers (0), p-Values and adjusted p-Values are also provided. (ZIP) [file pone.0160331.s008.zip › figuresDeSeq2 results design=Phenotype/mini.Unclassified_Faecalibacterium.png]

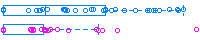

Supplement: S5 File — The degree of differential abundance is represented by log2 fold change (logFC) which indicates a positive or negative interaction (logFC >0 or <0) of the specified OTU in presence of Staphylococcus aureus. Plots representing the abundance of each OTU in the population of Staphylococcus aureus carriers (1) and non-carriers (0), p-Values and adjusted p-Values are also provided. (ZIP) [file pone.0160331.s008.zip › figuresDeSeq2 results design=Phenotype/mini.Unclassified_Helcococcus.png]

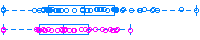

Supplement: S5 File — The degree of differential abundance is represented by log2 fold change (logFC) which indicates a positive or negative interaction (logFC >0 or <0) of the specified OTU in presence of Staphylococcus aureus. Plots representing the abundance of each OTU in the population of Staphylococcus aureus carriers (1) and non-carriers (0), p-Values and adjusted p-Values are also provided. (ZIP) [file pone.0160331.s008.zip › figuresDeSeq2 results design=Phenotype/mini.Unclassified_Lachnospiraceae.png]

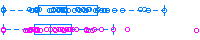

Supplement: S5 File — The degree of differential abundance is represented by log2 fold change (logFC) which indicates a positive or negative interaction (logFC >0 or <0) of the specified OTU in presence of Staphylococcus aureus. Plots representing the abundance of each OTU in the population of Staphylococcus aureus carriers (1) and non-carriers (0), p-Values and adjusted p-Values are also provided. (ZIP) [file pone.0160331.s008.zip › figuresDeSeq2 results design=Phenotype/mini.Unclassified_Oscillibacter.png]

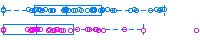

Supplement: S5 File — The degree of differential abundance is represented by log2 fold change (logFC) which indicates a positive or negative interaction (logFC >0 or <0) of the specified OTU in presence of Staphylococcus aureus. Plots representing the abundance of each OTU in the population of Staphylococcus aureus carriers (1) and non-carriers (0), p-Values and adjusted p-Values are also provided. (ZIP) [file pone.0160331.s008.zip › figuresDeSeq2 results design=Phenotype/mini.Unclassified_Prevotella.png]

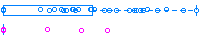

Supplement: S5 File — The degree of differential abundance is represented by log2 fold change (logFC) which indicates a positive or negative interaction (logFC >0 or <0) of the specified OTU in presence of Staphylococcus aureus. Plots representing the abundance of each OTU in the population of Staphylococcus aureus carriers (1) and non-carriers (0), p-Values and adjusted p-Values are also provided. (ZIP) [file pone.0160331.s008.zip › figuresDeSeq2 results design=Phenotype/mini.Unclassified_Roseburia.png]

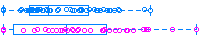

Supplement: S5 File — The degree of differential abundance is represented by log2 fold change (logFC) which indicates a positive or negative interaction (logFC >0 or <0) of the specified OTU in presence of Staphylococcus aureus. Plots representing the abundance of each OTU in the population of Staphylococcus aureus carriers (1) and non-carriers (0), p-Values and adjusted p-Values are also provided. (ZIP) [file pone.0160331.s008.zip › figuresDeSeq2 results design=Phenotype/mini.Unclassified_Vagococcus.png]

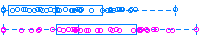

Supplement: S5 File — The degree of differential abundance is represented by log2 fold change (logFC) which indicates a positive or negative interaction (logFC >0 or <0) of the specified OTU in presence of Staphylococcus aureus. Plots representing the abundance of each OTU in the population of Staphylococcus aureus carriers (1) and non-carriers (0), p-Values and adjusted p-Values are also provided. (ZIP) [file pone.0160331.s008.zip › figuresDeSeq2 results design=Phenotype/mini.Unclassified_Wautersiella.png]

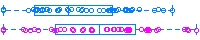

Supplement: S5 File — The degree of differential abundance is represented by log2 fold change (logFC) which indicates a positive or negative interaction (logFC >0 or <0) of the specified OTU in presence of Staphylococcus aureus. Plots representing the abundance of each OTU in the population of Staphylococcus aureus carriers (1) and non-carriers (0), p-Values and adjusted p-Values are also provided. (ZIP) [file pone.0160331.s008.zip › figuresDeSeq2 results design=Phenotype/mini.Vagococcus_fluvialis.png]

Pasteurella\_multocida Normalized Counts

$10^3$   
 $10^2$   
 $10^1$   
 $10^0$

AA

AG/GG

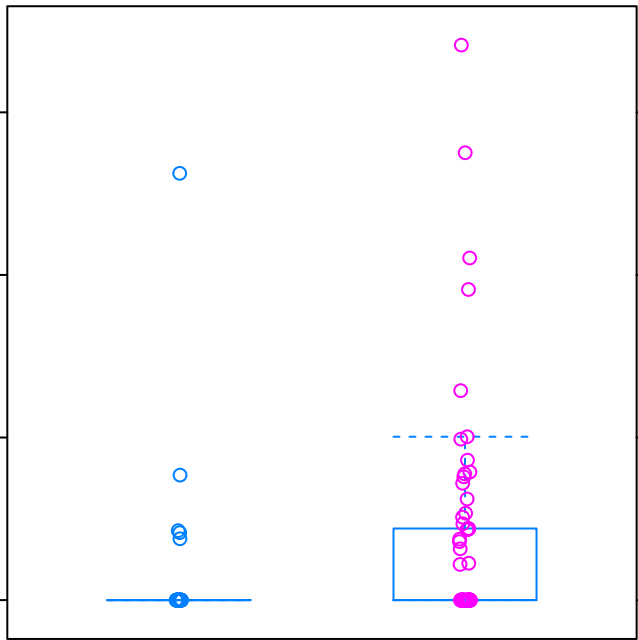

Supplement: S6 File — The degree of differential abundance is represented by log2 fold change (logFC) which indicates a positive or negative interaction (logFC >0 or <0) of the specified OTU in pigs with genotypes different than the non-carrier-associated genotype (AA). Plots representing the abundance of each OTU in the population of pigs with the non-carrier (AA) or other genotypes (AG/GG), p-Values and adjusted p-Values are also provided. (ZIP) [file pone.0160331.s009.zip › figuresDeSeq2 results design=Genotype/boxplot.Pasteurella_multocida.pdf]

Unclassified\_Klebsiella Normalized Counts

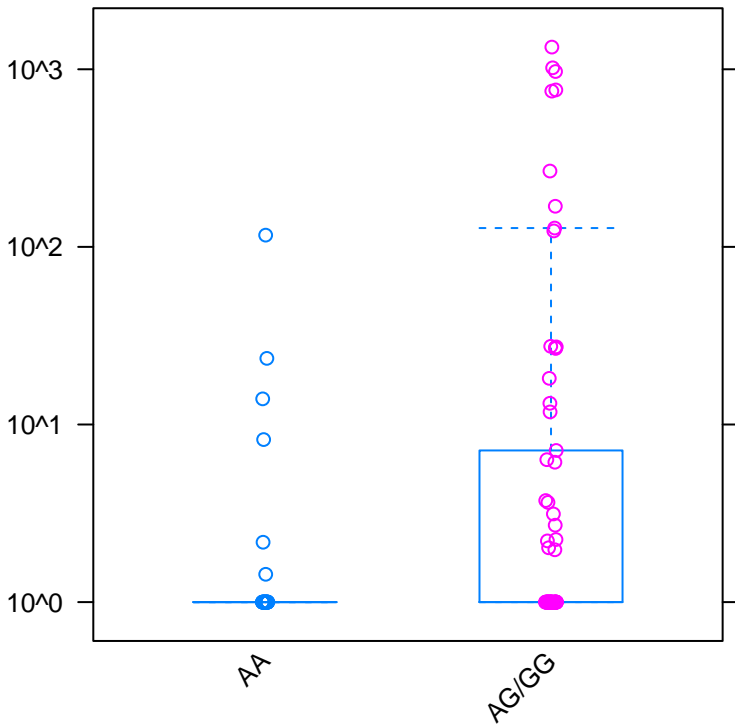

Supplement: S6 File — The degree of differential abundance is represented by log2 fold change (logFC) which indicates a positive or negative interaction (logFC >0 or <0) of the specified OTU in pigs with genotypes different than the non-carrier-associated genotype (AA). Plots representing the abundance of each OTU in the population of pigs with the non-carrier (AA) or other genotypes (AG/GG), p-Values and adjusted p-Values are also provided. (ZIP) [file pone.0160331.s009.zip › figuresDeSeq2 results design=Genotype/boxplot.Unclassified_Klebsiella.pdf]

Unclassified\_Lachnospiraceae Normalized Counts

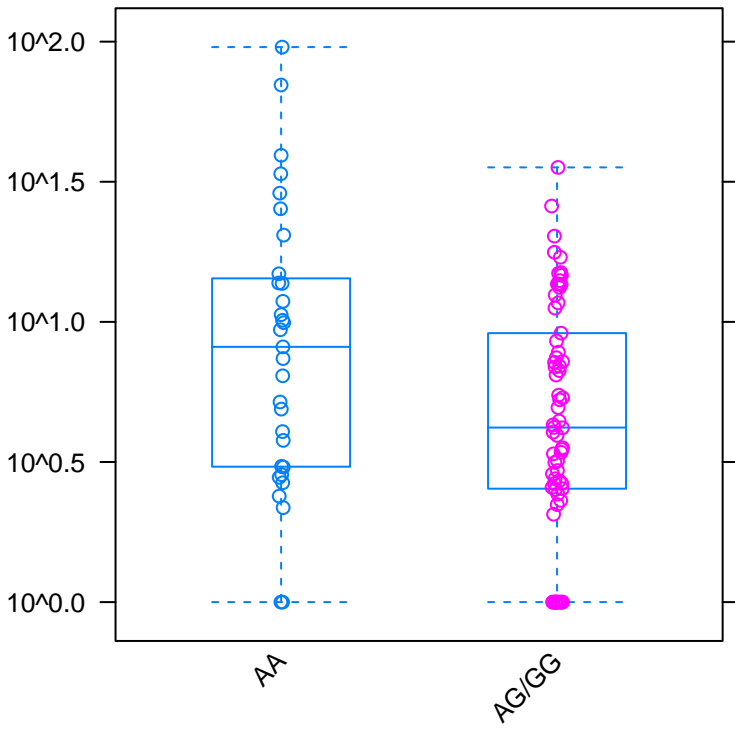

Supplement: S6 File — The degree of differential abundance is represented by log2 fold change (logFC) which indicates a positive or negative interaction (logFC >0 or <0) of the specified OTU in pigs with genotypes different than the non-carrier-associated genotype (AA). Plots representing the abundance of each OTU in the population of pigs with the non-carrier (AA) or other genotypes (AG/GG), p-Values and adjusted p-Values are also provided. (ZIP) [file pone.0160331.s009.zip › figuresDeSeq2 results design=Genotype/boxplot.Unclassified_Lachnospiraceae.pdf]

Unclassified\_Porphyrimonadaceae Normalized Counts

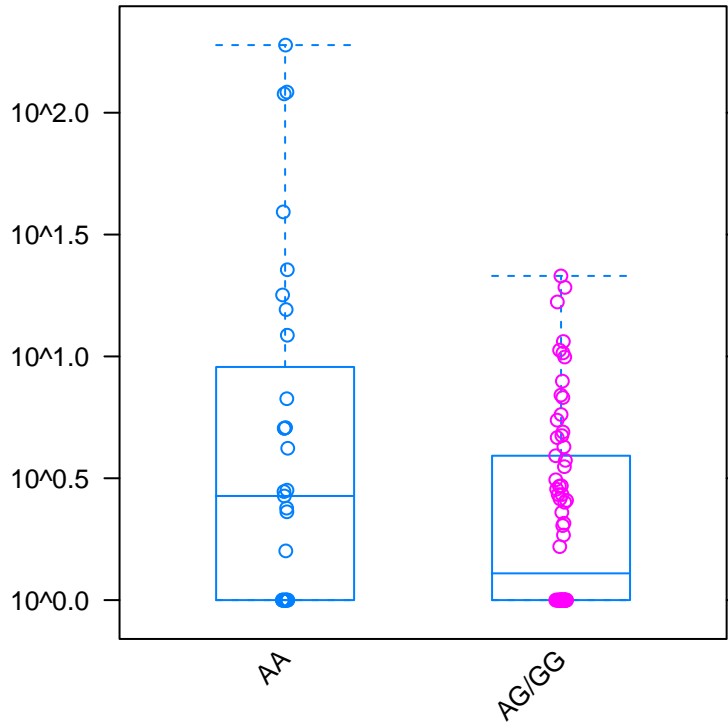

Supplement: S6 File — The degree of differential abundance is represented by log2 fold change (logFC) which indicates a positive or negative interaction (logFC >0 or <0) of the specified OTU in pigs with genotypes different than the non-carrier-associated genotype (AA). Plots representing the abundance of each OTU in the population of pigs with the non-carrier (AA) or other genotypes (AG/GG), p-Values and adjusted p-Values are also provided. (ZIP) [file pone.0160331.s009.zip › figuresDeSeq2 results design=Genotype/boxplot.Unclassified_Porphyromonadaceae.pdf]

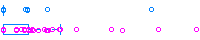

Supplement: S6 File — The degree of differential abundance is represented by log2 fold change (logFC) which indicates a positive or negative interaction (logFC >0 or <0) of the specified OTU in pigs with genotypes different than the non-carrier-associated genotype (AA). Plots representing the abundance of each OTU in the population of pigs with the non-carrier (AA) or other genotypes (AG/GG), p-Values and adjusted p-Values are also provided. (ZIP) [file pone.0160331.s009.zip › figuresDeSeq2 results design=Genotype/mini.Pasteurella_multocida.png]

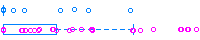

Supplement: S6 File — The degree of differential abundance is represented by log2 fold change (logFC) which indicates a positive or negative interaction (logFC >0 or <0) of the specified OTU in pigs with genotypes different than the non-carrier-associated genotype (AA). Plots representing the abundance of each OTU in the population of pigs with the non-carrier (AA) or other genotypes (AG/GG), p-Values and adjusted p-Values are also provided. (ZIP) [file pone.0160331.s009.zip › figuresDeSeq2 results design=Genotype/mini.Unclassified_Klebsiella.png]

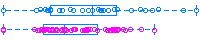

Supplement: S6 File — The degree of differential abundance is represented by log2 fold change (logFC) which indicates a positive or negative interaction (logFC >0 or <0) of the specified OTU in pigs with genotypes different than the non-carrier-associated genotype (AA). Plots representing the abundance of each OTU in the population of pigs with the non-carrier (AA) or other genotypes (AG/GG), p-Values and adjusted p-Values are also provided. (ZIP) [file pone.0160331.s009.zip › figuresDeSeq2 results design=Genotype/mini.Unclassified_Lachnospiraceae.png]

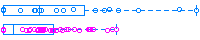

Supplement: S6 File — The degree of differential abundance is represented by log2 fold change (logFC) which indicates a positive or negative interaction (logFC >0 or <0) of the specified OTU in pigs with genotypes different than the non-carrier-associated genotype (AA). Plots representing the abundance of each OTU in the population of pigs with the non-carrier (AA) or other genotypes (AG/GG), p-Values and adjusted p-Values are also provided. (ZIP) [file pone.0160331.s009.zip › figuresDeSeq2 results design=Genotype/mini.Unclassified_Porphyromonadaceae.png]
