# Supplementary material for: Differential Analysis of the Nasal Microbiome of Pig Carriers or Non-Carriers of Staphylococcus aureus
Source: PLoS One. 2016 Aug 10;11(8):e0160331. doi: 10.1371/journal.pone.0160331 (PMC4980049; doi:10.1371/journal.pone.0160331)
Supplement: S5 File — The degree of differential abundance is represented by log2 fold change (logFC) which indicates a positive or negative interaction (logFC >0 or <0) of the specified OTU in presence of Staphylococcus aureus. Plots representing the abundance of each OTU in the population of Staphylococcus aureus carriers (1) and non-carriers (0), p-Values and adjusted p-Values are also provided. (ZIP) [file pone.0160331.s008.zip › DeSeq2 results design=Phenotype.html]

Differentially abundant OTUs between pigs phenotypically classified as <i>Staphylococcus aureus</i> carriers (1) or non-carriers (0)


## Differentially abundant OTUs between pigs phenotypically classified as *Staphylococcus aureus* carriers (1) or non-carriers (0)

| ID | Image | logFC | p-Value | Adjusted p-Value |
| --- | --- | --- | --- | --- |
| ID | Image | logFC | p-Value | Adjusted p-Value |
| Unclassified\_Anaerococcus |  | -3.33 | 1.03e-08 | 2.19e-06 |
| Kurthia\_gibsonii |  | -3.49 | 1.88e-08 | 2.19e-06 |
| Unclassified\_Helcococcus |  | -2.73 | 2.45e-06 | 1.89e-04 |
| Unclassified\_Roseburia |  | -3.09 | 8.49e-06 | 4.93e-04 |
| Unclassified\_Wautersiella |  | 2.49 | 3.17e-05 | 1.47e-03 |
| Moraxella\_boevrei |  | -2.62 | 1.17e-04 | 4.54e-03 |
| Unclassified\_Lachnospiraceae |  | -1.21 | 3.11e-04 | 1.03e-02 |
| Unclassified\_Oscillibacter |  | -1.30 | 3.70e-04 | 1.06e-02 |
| Unclassified\_Prevotella |  | -1.60 | 4.37e-04 | 1.06e-02 |
| Unclassified\_Facklamia |  | -1.47 | 4.82e-04 | 1.06e-02 |
| Acinetobacter\_lwoffii |  | -2.22 | 5.02e-04 | 1.06e-02 |
| Anaerococcus\_lactolyticus |  | -3.22 | 5.53e-04 | 1.07e-02 |
| Unclassified\_Faecalibacterium |  | -1.09 | 1.07e-03 | 1.92e-02 |
| Unclassified\_Vagococcus |  | 1.56 | 1.24e-03 | 2.05e-02 |
| Vagococcus\_fluvialis |  | 1.54 | 2.40e-03 | 3.25e-02 |
| Leuconostoc\_pseudomesenteroides |  | -2.38 | 2.45e-03 | 3.25e-02 |
| Unclassified\_Acinetobacter |  | -1.63 | 2.48e-03 | 3.25e-02 |
| Facklamia\_tabacinasalis |  | -1.78 | 2.52e-03 | 3.25e-02 |
| Leuconostoc\_mesenteroides |  | -1.98 | 3.30e-03 | 4.03e-02 |
| Unclassified\_Aerococcaceae |  | -1.90 | 3.60e-03 | 4.06e-02 |
| Acinetobacter\_soli |  | -2.84 | 3.67e-03 | 4.06e-02 |
| Unclassified\_Chitinophagaceae |  | -2.84 | 4.43e-03 | 4.67e-02 |

| ID | Image | logFC | p-Value | Adjusted p-Value |
| --- | --- | --- | --- | --- |

(Page generated on Tue Mar 15 17:48:59 2016 by ReportingTools 2.10.0 and hwriter )
