# Supplementary material for: Differential Analysis of the Nasal Microbiome of Pig Carriers or Non-Carriers of Staphylococcus aureus
Source: PLoS One. 2016 Aug 10;11(8):e0160331. doi: 10.1371/journal.pone.0160331 (PMC4980049; doi:10.1371/journal.pone.0160331)
Supplement: S6 File — The degree of differential abundance is represented by log2 fold change (logFC) which indicates a positive or negative interaction (logFC >0 or <0) of the specified OTU in pigs with genotypes different than the non-carrier-associated genotype (AA). Plots representing the abundance of each OTU in the population of pigs with the non-carrier (AA) or other genotypes (AG/GG), p-Values and adjusted p-Values are also provided. (ZIP) [file pone.0160331.s009.zip › DeSeq2 results design=Genotype.html]

Differentially abundant OTUs between pigs classified by their <i>Staphylococcus aureus</i> carriage-associated genotypes as carriers (AG/GG) or non-carriers (AA)


## Differentially abundant OTUs between pigs classified by their *Staphylococcus aureus* carriage-associated genotypes as carriers (AG/GG) or non-carriers (AA)

| ID | Image | logFC | p-Value | Adjusted p-Value |
| --- | --- | --- | --- | --- |
| ID | Image | logFC | p-Value | Adjusted p-Value |
| Unclassified\_Porphyromonadaceae |  | -2.69 | 6.11e-06 | 0.00107 |
| Unclassified\_Lachnospiraceae |  | -1.25 | 4.04e-04 | 0.02600 |
| Pasteurella\_multocida |  | 3.24 | 4.45e-04 | 0.02600 |
| Unclassified\_Klebsiella |  | 3.37 | 6.53e-04 | 0.02860 |

| ID | Image | logFC | p-Value | Adjusted p-Value |
| --- | --- | --- | --- | --- |

(Page generated on Tue Mar 15 17:51:58 2016 by ReportingTools 2.10.0 and hwriter )
